# A Bilingual Benchmark for Evaluating Diagnostic Performance of Multimodal Large Language Models in Radiology (RadM-Bench): Evaluation Development and Validation

**DOI:** 10.2196/92183

**Published:** 2026-08-07

**Authors:** Qingxia Wu, Qingxia Wu, Peipei Zhang, Zhifeng Yi, Yu Shen, Yan Bai, Hongna Tan, Pei Dong, Zhong Xue, Neil Roberts, Meiyun Wang

**Affiliations:** 1 Department of Medical Imaging, Henan Provincial People’s Hospital & People’s Hospital of Zhengzhou University, No.7 Weiwu Road, Zhengzhou, Henan, 450003, China, 86 037165580267, 86 037165651056; 2 Department of Research and Cooperation, United Imaging Intelligence, Beijing, Beijing, China; 3 Department of Nuclear Medicine, Hangzhou Cancer Hospital, Hangzhou, Zhejiang, China; 4 Emergency Department, Huangshi Central Hospital, Huangshi, Hubei, China; 5 Department of Research and Cooperation, United Imaging Intelligence, Shanghai, Shanghai, China; 6 Biomedical Research Institute, Henan Academy of Sciences, Zhengzhou, Henan, China; 7 Center for Reproductive Health, Institute for Regeneration and Repair, University of Edinburgh, Edinburgh, Scotland, United Kingdom

**Keywords:** benchmark, multimodal, large language model, radiology, diagnosis

## Abstract

**Background:**

Multimodal large language models are increasingly used in radiological diagnosis, but their performance has not been systematically evaluated across volumetric (3D) imaging, real-world clinical versus public teaching cases, and bilingual contexts.

**Objective:**

The aim of the study is to develop a bilingual radiology benchmark and characterize the diagnostic performance of state-of-the-art multimodal large language models across input modality, clinical setting (public teaching vs routine clinical), disease rarity, and clinical-history language and to disentangle linguistic from clinical-content effects through a cross-linguistic control experiment.

**Methods:**

We constructed RadM-Bench, comprising 720 cases evenly distributed across 9 radiological subspecialties: 360 English public teaching cases enriched in rare diseases (RadEdu) and 360 Chinese routine clinical cases (RealClin). In total, 4 proprietary models (GPT-4o, O3, Gemini-2-Flash, and Gemini-2.5-Flash-Thinking) and 6 open-source models (Qwen2.5-VL-72B/7B, InternVL3-78B/8B, Llama-4-Scout-17B-16E, and MedGemma-4B) were evaluated under 4 input conditions: clinical history alone, history with radiologist-selected 2D key images, and history with volumetric data sampled at 2 and 10 frames per second (fps). Each response was scored on a 4-tier 0‐3 diagnostic-quality rubric by 2 board-certified radiologists blinded to model identity. Mean scores with bias-corrected and accelerated bootstrap 95% CIs are reported. To disentangle language from clinical content, all 360 RealClin histories were translated into English and re-evaluated, with paired comparisons by Wilcoxon signed-rank tests and Benjamini-Hochberg false-discovery-rate correction.

**Results:**

Mean performance remained below 1.5 on the 0‐3 scale for all 10 models on both datasets. Adding radiologist-selected 2D key images to clinical history improved performance in all 10 models (+19.8% to +139.2%). In RealClin at fps=10, all 8 evaluable models scored lower with volumetric input than with the 2D-image baseline (−5.3% to −31.4%); MedGemma-4B and Llama-4-Scout-17B-16E could only be evaluated at fps=2 due to context-window and graphics processing unit–memory constraints. At fps=2, a total of 8 out of 10 models declined (−6.8% to −28.6%), while Qwen2.5-VL-7B and InternVL3-8B showed marginal improvements (+2.4% and +2.1%). Cross-dataset transfer diverged by model category: proprietary models declined from RadEdu to RealClin (eg, O3 with images: 1.14 to 0.79), whereas Chinese-centric open-source models improved (eg, InternVL3-78B: 0.48 to 0.75). The rare-disease premium observed in 9 out of 10 models in RadEdu reversed in RealClin, where common-disease scores exceeded rare-disease scores in 7 out of 10 models under history-only input. Translating RealClin histories into English produced a numerical decrease in mean score for all 10 models, which were statistically significant in 9 out of 10 models after false discovery rate correction, excluding a Chinese-language penalty.

**Conclusions:**

Within the scope of this benchmark, multimodal inputs improved performance over clinical history alone, but performance gaps remain in volumetric data processing and cross-context generalization, with mean diagnostic performance across the 10 evaluated models remaining below clinically actionable levels on both datasets.

## Introduction

Radiological diagnosis unfolds through progressive information integration. Physicians use patient history to select appropriate imaging modalities such as computed tomography (CT) or magnetic resonance imaging (MRI) [1]. These multislice volumetric images provide comprehensive 3D information for evaluating anatomical structures and tissue distinctiveness, which radiologists then extract into representative 2D key images to aid diagnosis [2]. However, most educational resources and by extension many AI benchmarks rely on curated 2D snapshots [3-5], whereas clinical practice requires full 3D analysis. It remains uncertain whether AI can maintain accuracy with escalating modal complexity, creating a gap between benchmark performance and real-world utility.

Multimodal large language models (MLLMs) show promise in medical image interpretation; yet, comprehensive evaluation reveals substantial performance limitations and systematic assessment gaps [5-7]. The rapid diversification of MLLM architectures has outpaced systematic comparative evaluation across clinical contexts [8]. While individual models show promise in controlled settings, accuracy varies substantially across architectures and clinical scenarios, with performance gains from multimodal input plateauing below expert thresholds and model rankings shifting unpredictably [5,6]. These architectural paradigms entail complex trade-offs across diagnostic accuracy, computational requirements, data privacy, and deployment flexibility that remain poorly characterized in medical settings. While computational efficiency, privacy preservation, and deployment flexibility represent important directions for future research, this work focuses squarely on establishing a rigorous bilingual benchmark (RadM-Bench) to enable standardized, reproducible assessment of diagnostic performance, a critical first step toward evidence-based model selection for clinical radiology.

Current evaluation frameworks exhibit 5 critical gaps. First, existing benchmarks rely predominantly on static 2D images, underrepresenting 3D volumetric data essential to radiological practice and spatial reasoning [2,9-11]. Second, evaluation datasets frequently use publicly available teaching collections that may overlap with the web-scale corpora used to pretrain general-purpose MLLMs, raising the possibility that performance on such datasets is inflated by prior exposure rather than reflecting genuine generalization [4,10-12]. Third, published evaluations are linguistically homogeneous, being conducted mostly on English-language datasets [11,13]. Fourth, benchmarks exhibit systematic epidemiological bias toward rare or pedagogically valuable pathologies that maximize educational impact but poorly represent routine clinical disease prevalence patterns [11,12], creating a misrepresentative “rarity premium” and overestimating clinical utility [3]. Fifth, current assessments depend on simplistic binary correctness metrics or Likert scales (0‐5 scoring) [14-16], failing to capture the hierarchical nature of diagnostic reasoning and the graduated utility of partially correct diagnoses in clinical management [3]. Addressing these gaps is essential to determine whether model performance degrades when faced with volumetric studies, routine disease spectra, and non-English clinical contexts, while using more sophisticated assessment frameworks that reflect the nuanced hierarchy of clinical diagnostic accuracy.

To address these evaluation gaps and systematically assess MLLMs across diverse health care contexts, we developed RadM-Bench, a comprehensive bilingual benchmark comprising 720 radiology cases evenly distributed across 9 subspecialties. Adopting a more reasonable methodological framework enables comprehensive comparison across architectural paradigms, input complexity, linguistic contexts, and disease prevalence distributions to determine whether current MLLMs demonstrate robust clinical utility beyond controlled educational scenarios.

## Methods

### Dataset Construction and Composition

The RadM-Bench dataset was constructed from 2 complementary data sources to enable comprehensive evaluation of multimodal diagnostic capabilities across educational and routine clinical scenarios, comprising 720 multimodal studies evenly distributed across 9 radiological subspecialties (abdominal, breast, cardiovascular, chest, head and neck, musculoskeletal, neuroradiology, pediatric, and urogenital imaging).

- RadEdu (n=360): The RadEdu comprised educational cases systematically selected from Teaching Cases in Eurorad between August 16, 1999, and May 15, 2024 [17]. Inclusion criteria required at least 1 image from conventional radiography, CT, or MRI. Cases were excluded (1) if they exclusively involved ultrasound, nuclear medicine, or digital subtraction angiography, contained only nonradiological images, or (2) if they exceeded 16 images to maintain computational feasibility. From 9640 eligible studies, a stratified random sample of 360 cases was drawn with 40 cases per subspecialty. Each case included radiologist-selected 2D images paired with English clinical histories, with rare disease status classified according to the National Organization for Rare Disorders (NORD) catalog [18].
- RealClin (n=360): The RealClin comprised routine examinations from a Chinese academic tertiary hospital between January 1, 2022, and January 1, 2024. Board-certified radiologists retrospectively and randomly selected cases from daily practice, applying the same modality criteria and subspecialty-balanced sampling described earlier. For each case, 2 complementary visual representations were prepared to support evaluation under both the 2D-image and the volumetric input conditions.

For the 2D condition, the supervising radiologist prospectively selected the slices considered diagnostically informative for that case (mirroring the slice-curation step that radiologists routinely perform when preparing teaching files or report illustrations). Selected slices were exported as static PNG or JPEG key images at the clinically appropriate window and level for the relevant pathology.

For the volumetric condition, complete CT and MRI series were prepared as follows. The supervising radiologist first reviewed each Digital Imaging and Communications in Medicine (DICOM) series in RadiAnt DICOM Viewer (Medixant) and selected the window and level settings most appropriate for visualizing the relevant pathology (eg, lung window for pulmonary lesions, mediastinal window for cardiac structures, and narrow window for liver lesions). The series was then exported once with fixed parameters (512×512 pixel spatial resolution, 10 frames per second [fps], “Best” movie quality, and H.264 High Profile) to produce a single canonical MP4 cine clip that served as the volumetric representation for that case. For models that subsequently required sequential image inputs rather than native video, frames were extracted from this same MP4 file using OpenCV (cv2.VideoCapture) without any additional re-encoding, ensuring that all models processed pixel data derived from the same clinician-curated source. Multisequence MRI and multiphase CT acquisitions were stored as separate MP4 files per sequence or phase to preserve their synergistic diagnostic information. Per-model implementation of frame sampling and dispatch to video or frame-stack input is detailed in the Volumetric Data Processing and Frame Sampling section.

Each Chinese clinical history was paired with the corresponding case. Rare disease classification followed the Chinese National Health Commission’s Rare Disease Directory [19,20], and all data underwent deidentification through DICOM tag removal and manual redaction.

### Model Selection and Configuration

We evaluated 10 state-of-the-art MLLMs to encompass the present spectrum of proprietary versus open-source development, parameter scale, and language orientation. Proprietary systems were accessed via their production APIs: gemini-2.0-flash, gemini-2.5-flash-preview-04-17-thinking [21], gpt-4o-2024-11-20, and o3, representing both nonreasoning- and reasoning-enhanced commercial offerings [22]. Open-source models were executed locally on NVIDIA H20 141 GB graphics processing units (GPUs) and stratified by parameter count: large models comprising Qwen2.5-VL-72B (Alibaba, Chinese-centric) [23], InternVL3-78B (Shanghai AI Lab, Chinese-centric) [24], and Llama-4-Scout-17B-16E (Meta, English-centric) [25]; and smaller models consisting of Qwen2.5-VL-7B [26], InternVL3-8B [27], and MedGemma-4B (Google) [28]. For brevity, figures and tables refer to each model by its lowercase technical identifier (eg, qwen2.5-vl-72b).

Each model was queried under four progressively enriched input conditions: (1) text-only using clinical history alone, (2) text plus radiologist-curated key images for all 720 cases, (3) text plus volumetric images sampled at 2 fps, and (4) text plus volumetric images sampled at 10 fps; conditions (3) and (4) were applied to the 360 RealClin cases. Generation parameters were standardized across all models (temperature=0, top-k=1, top-p=1; remaining settings at default) to ensure reproducible comparability. For the O3 model, all generation parameters were set to the official API default values. Consistent with its design as a reasoning model, traditional parameters such as temperature, top-k, and top-p were not applicable or supported in the API calls and thus were not specified. The reasoning_effort parameter was also not explicitly set and used its default tier. Standardized prompts in English and Chinese requested exactly 5 ranked differential diagnoses in strict JSON format without additional explanations, with system prompts constraining outputs to concise medical diagnoses based on provided clinical information and visual inputs. Detailed prompts are provided in Multimedia Appendix 1.

### Volumetric Data Processing and Frame Sampling

Volumetric studies were evaluated under 2 temporal sampling conditions: a full-rate condition at 10 fps (all original frames provided) and a subsampled condition at 2 fps (uniformly subsampled frames). For models accepting native video input, MP4 files were supplied directly; for models requiring frame stacks, frames were pre-extracted as JPEGs at the corresponding rate. MedGemma-4B and Llama-4-Scout-17B-16E could not be evaluated at fps=10 due to context-window and GPU-memory constraints, and results for these 2 models are therefore reported only at fps=2. Per-model implementation, tokenization, and token-budget verification are detailed in Multimedia Appendix 1.

### Evaluation Framework and Scoring Methodology

For publicly available cases from the Eurorad, the reference diagnosis was defined as the final diagnosis provided by the Eurorad platform. For the RealClin, reference diagnoses were established using the following evidence-based criteria. Histopathological results from biopsy or surgical resection served as the gold standard for lesions with available specimens, such as pulmonary adenocarcinoma. Congenital anomalies and characteristic morphological findings definitively diagnosed by imaging alone, including aneurysm, ventricular septal defect, and Budd-Chiari syndrome, were confirmed by senior radiologists according to standard radiological criteria. Inflammatory, infectious, or functional conditions were diagnosed based on images, laboratory results, or clinical discharge summaries, such as small bowel angioedema secondary to C1-esterase-inhibitor deficiency, encephalitis, or cardiomyopathy.

MLLM evaluation used a structured JSON-formatted prompt requiring 5 differential diagnoses ranked by clinical likelihood. For every benchmark case, models were prompted in their native language (English for RadEdu and Chinese for RealClin) with standardized instructions requesting exactly 5 differential diagnoses ranked from most- to least-likely and returned in a predefined JSON schema (detailed prompts in Multimedia Appendix 1). All models were instructed to produce outputs in strict JSON, and most responses complied. Infrequent cases of invalid JSON output were manually reviewed, parsed, and standardized before grading to ensure consistent evaluation.

**Figure 1.**
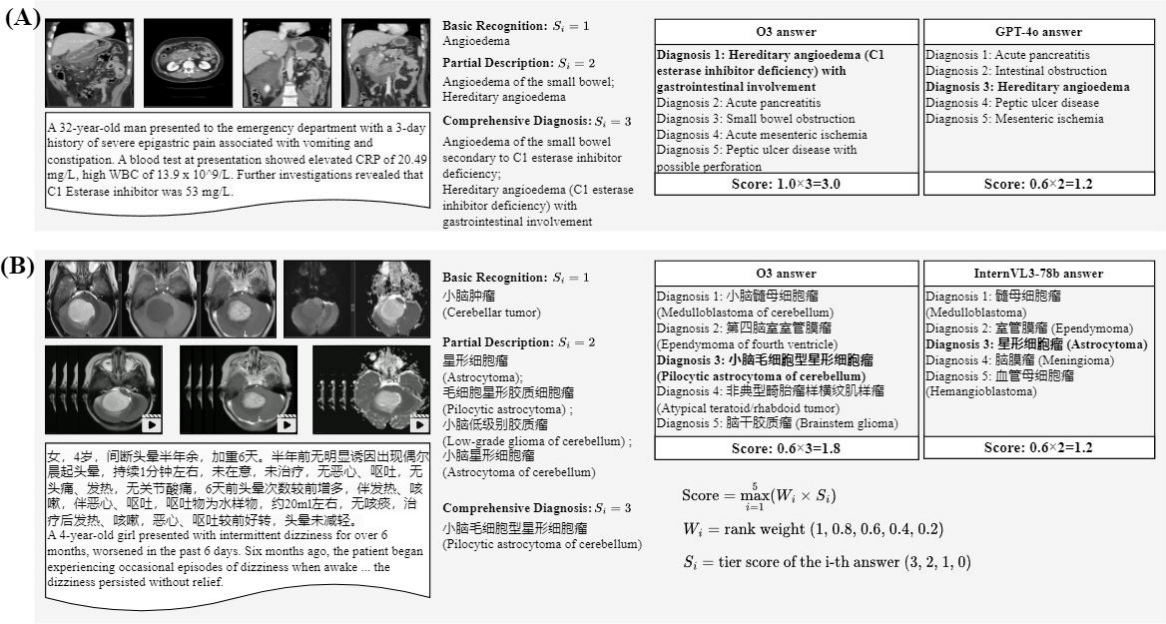
Representative cases and scoring methodology. (A) English RadEdu case example. (B) Chinese RealClin case example. For visual clarity and readability, the model outputs presented here are formatted as plain-text representations of the parsed JSON responses generated by each model.

This approach ensured consistent parsing and evaluation across all model responses. The scoring framework incorporated 3 hierarchical levels reflecting diagnostic completeness: basic recognition (1 point) for fundamental identification of the disease class (eg, “angioedema”), partial description (2 points) for partially specific diagnosis combining correct pathology with either organ localization or etiology (eg, “angioedema of the small bowel” or “hereditary angioedema”), and comprehensive diagnosis (3 points) for complete formulation integrating both localization and causative mechanism (eg, “hereditary angioedema with small-bowel involvement due to C1-esterase-inhibitor deficiency”). To simulate clinical decision-making priorities and quantify the real-world clinical utility of ranked diagnoses, position-dependent weights were applied to each ranked diagnosis (1.0, 0.8, 0.6, 0.4, and 0.2 for positions 1‐5, respectively). This sequential weight decay reflects the standard of radiology care, where top-ranked diagnoses directly drive initial patient management and carry the highest actionable value, while lower-ranked diagnoses represent low-probability alternatives with limited clinical impact. This structure also methodologically prevents inflated performance from indiscriminate “shotgun” differential listing, ensuring that we measure true diagnostic stratification ability rather than simple disease recall. The final performance score was calculated as the maximum product of diagnostic quality score and positional weight across all 5 responses (score=max(*W*_*i*_×*S*_*i*_), where *W*_*i*_ represents rank weight, and *S*_*i*_ represents tier score). This max-based formulation is grounded in the core clinical competency of placing the correct diagnosis in an appropriately high-probability rank: a correct diagnosis in a prioritized position is sufficient to guide appropriate clinical care, even if other lower-probability differentials are incorrect. This approach acknowledges that accurate diagnosis anywhere within the differential demonstrates clinical competency, avoids unfair penalty for reasonable low-probability differentials, and appropriately rewards higher-ranked accurate diagnoses and more comprehensive diagnostic formulation. Representative scoring examples are illustrated in Figure 1.

In addition to the max(*W*_*i*_*×S*_*i*_) performance score, we computed top-1, top-3, and top-5 accuracy as complementary metrics. Top-*k* accuracy was defined as the proportion of cases for which any candidate diagnosis at tier *S*_*i*_=1, 2, or 3 (basic recognition, partial description, or comprehensive diagnosis) appeared within the first *k* ranked diagnoses produced by the model.

### Diagnostic Quality Grading

Both graders (2 board-certified radiologists) were blinded to model identities throughout the grading process. Model outputs were stripped of all model-identifying metadata, randomly shuffled, and presented through a custom web-based grading interface that displayed only the clinical history, reference diagnosis, and the candidate differential diagnoses to be graded. Interrater reliability was established during a prespecified calibration phase prior to full grading. Both graders independently graded a randomly selected subset of 50 cases drawn from the 720-case pool. Cohen κ on this calibration set was 0.78, indicating substantial agreement [29], and exceeded the prespecified threshold of κ≥0.75. The 2 graders then proceeded to grade the full dataset and resolved disagreements through consensus review with reference to the rubric.

### Cross-Linguistic Control Experiment

To assess whether the language of the clinical history (Chinese in RealClin vs English in RadEdu) acted as a confounding variable in the cross-dataset comparison, we conducted a paired cross-linguistic control experiment on the RealClin subset. The clinical history of every RealClin case was translated from Chinese into English using DeepSeek-V3 (deepseek-chat, accessed via the official API in April 2026). Each of the 10 models was then re-evaluated on the translated English version under identical experimental settings (same prompt template, same generation parameters, and same diagnostic-quality grading).

### Statistics and Reproducibility

All analyses were performed using R statistical software (version 4.1.2; R Foundation for Statistical Computing). Mean performance scores (0‐3 scale) and 95% CIs were calculated using the bias-corrected and accelerated bootstrap percentile method with 2000 resamples. Accuracy metrics (top-1, top-3, and top-5) and their 95% CIs were calculated using the Clopper-Pearson exact method for binomial proportions. Paired within-model comparisons between original Chinese and translated English clinical histories in RealClin were performed using the 2-tailed Wilcoxon signed rank test (n=360 paired cases per model). Comparisons of group proportions in dataset composition were performed using 2-sided *z* tests. To control the false discovery rate across the 10 cross-linguistic comparisons, *P* values were adjusted using the Benjamini-Hochberg (BH) procedure with *q*=0.05; both raw and BH-adjusted *P* values are reported. Significance was assessed at α=.05.

### Ethical Considerations

This study was approved by the institutional review board of Henan Provincial People’s Hospital (approval: 2025163) and conducted in line with the Declaration of Helsinki. It included retrospective deidentified clinical data and publicly available educational radiology cases for secondary analysis. Informed consent was waived by the ethics committee due to the retrospective nature and full anonymization of all data, which ensured participant privacy and confidentiality. No compensation was provided to participants, and all images in the paper and multimedia appendices are anonymized with no identifiable individual features.

## Results

### Dataset Characteristics and Construction

**Figure 2.**
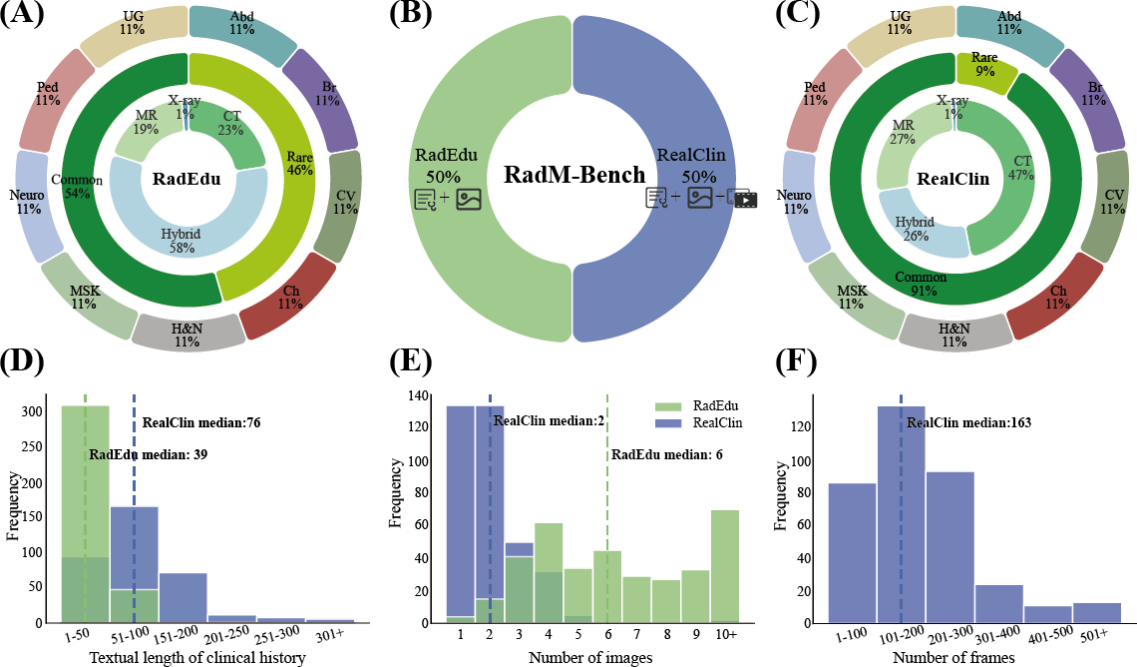
Dataset characteristics and construction of RadM-Bench. (A) RadEdu subset composition showing subspecialty distribution (outer ring), disease rarity classification (middle ring), and modality distribution (inner ring). (B) Overall RadM-Bench structure with RadEdu (n=360, 50%) and RealClin (n=360, 50%) subsets, illustrating the bilingual English-Chinese composition. (C) RealClin subset composition showing subspecialty distribution (outer ring), disease rarity classification (middle ring), and modality distribution (inner ring). (D) Distribution of clinical history text length measured in words. (E) Number of key images per case. (F) Volumetric data representation showing number of frames per case. Abd: abdominal; Br: breast; Ch: chest; CT: computed tomography; CV: cardiovascular; H&N: head and neck; MR: magnetic resonance; MSK: musculoskeletal; Neuro: neurological; Ped: pediatric; UG: urogenital; X-ray: radiography.

The RadM-Bench dataset comprises 720 multimodal medical imaging cases uniformly distributed across 9 radiological subspecialties, with 80 cases per subspecialty (Figure 2A-C). It consists of 2 distinct subsets: RadEdu (n=360) and RealClin (n=360).

Significant compositional differences existed between the 2 subsets, highlighting the distinct characteristics of educational versus real-world clinical data (Table 1). RadEdu predominantly featured hybrid imaging (207/360, 57.5%), followed by CT (81/360, 22.5%) and magnetic resonance (MR; 67/360, 18.6%), whereas RealClin showed a higher proportion of CT (169/360, 46.9%) and MR (96/360, 26.7%) studies, with fewer hybrid cases (92/360, 25.6%). Disease rarity differed markedly: RadEdu had a substantially higher proportion of rare diseases (164/360, 45.6% vs 31/360, 8.6%; *P*<.001), reflecting the educational emphasis on diagnostically challenging or pedagogically valuable cases, while RealClin predominantly comprised common diseases (329/360, 91.4%) representative of routine clinical practice.

Clinical information presentation varied significantly between subsets (Figure 2D-F). RealClin cases contained more comprehensive clinical histories (median 76, IQR 45‐106 vs median 39, IQR 25‐49 words; *P*<.001) but fewer images (median 2, IQR 1‐3 vs median 6, IQR 4‐9 images; *P*<.001). RealClin additionally included video data derived from volumetric CT or MR acquisitions (median 163, IQR 105‐242 frames; median 2, IQR 1‐6 videos), enabling comparison between curated key images and comprehensive imaging data for multimodal model evaluation.

The linguistic diversity of RadM-Bench, with RadEdu cases presented entirely in English and RealClin in Chinese, enhances its utility for cross-cultural evaluation. Because the RealClin cases were drawn from an internal hospital database that was not publicly released, the likelihood of direct exposure during MLLM pretraining is reduced relative to public test sets, although exposure of similar regional clinical data during open-source model pretraining cannot be excluded; this enables a more rigorous, although not contamination-free, assessment of model generalization in real-world clinical scenarios.

**Table 1.** Baseline characteristics of RadM-Bench dataset.

	RadEdu (n=360)	RealClin (n=360)	All (N=720)
Modality, n (%)			
Computed tomography	81 (22.5)	169 (46.9)	250 (34.7)
Hybrid	207 (57.5)	92 (25.6)	299 (41.5)
Magnetic resonance	67 (18.6)	96 (26.7)	163 (22.6)
Radiography	5 (1.4)	3 (0.8)	8 (1.1)
Subspecialty, n (%)			
Abdominal	40 (11.1)	40 (11.1)	80 (11.1)
Breast	40 (11.1)	40 (11.1)	80 (11.1)
Cardiovascular	40 (11.1)	40 (11.1)	80 (11.1)
Chest	40 (11.1)	40 (11.1)	80 (11.1)
Head and neck	40 (11.1)	40 (11.1)	80 (11.1)
Musculoskeletal	40 (11.1)	40 (11.1)	80 (11.1)
Neuroradiology	40 (11.1)	40 (11.1)	80 (11.1)
Pediatric	40 (11.1)	40 (11.1)	80 (11.1)
Urogenital	40 (11.1)	40 (11.1)	80 (11.1)
Disease rarity, n (%)			
Common disease	196 (54.4)	329 (91.4)	525 (72.9)
Rare disease	164 (45.6)	31 (8.6)	195 (27.1)
Text length of clinical history, median (IQR)	39 (25-49)	76 (45-106)	49 (27-79)
Images, median (IQR)	6 (4-9)	2 (1-3)	3 (2-6)
Frames, median (IQR)	—^a^	163 (105-242)	—
Videos, median (IQR)	—	2 (1-6)	—

a Not applicable.

### Overall Performance Patterns Across Input Modalities

**Figure 3.**
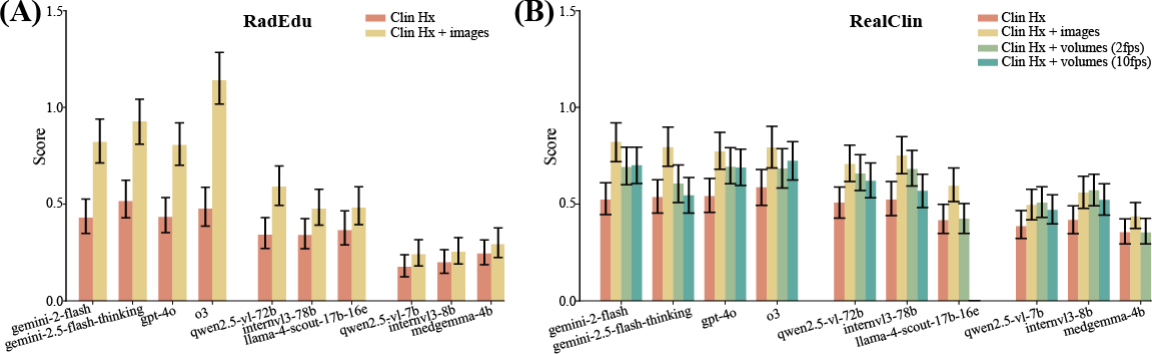
Model performance across input modalities and datasets. (A) Performance comparison of 10 multimodal large language models in RadEdu cases under 2 input conditions: clinical history only (Clin Hx, red) and clinical history plus key images (Clin Hx+images, yellow). (B) Corresponding performance in RealClin cases under 4 input conditions: clinical history only (Clin Hx, red), clinical history plus key images (Clin Hx+images, yellow), clinical history plus volumetric data sampled at 2 fps (Clin Hx+volumes 2fps, light teal), and clinical history plus volumetric data at 10 fps (Clin Hx+volumes 10 fps, dark teal). llama-4-scout-17b-16e and medgemma-4B were evaluated only at fps=2 due to context-window and graphics processing unit–memory constraints. Error bars represent 95% CIs.

Evaluation across both datasets revealed that all models achieved limited performance (scores<1.5), indicating substantial room for improvement in multimodal medical diagnosis (Figure 3 and Figure S1 and Tables S1-S4 in Multimedia Appendix 2). Despite these limitations, multimodal inputs consistently surpassed clinical history alone, while modality-specific effects varied by model architectures. Integrating radiologist-curated key images with clinical history provided substantial diagnostic improvements across all models in both RadEdu and RealClin datasets. In RadEdu, proprietary models showed the most substantial improvements, with O3 achieving the highest performance advancement from 0.48 (95% CI 0.39-0.59) to 1.14 (95% CI 1.02-1.28; Δ=+0.66, +139.2%). Large open-source models demonstrated more moderate yet consistent gains, with Qwen2.5-VL-72B showing the largest gain (mean 0.34, 95% CI 0.27-0.43 to mean 0.59, 95% CI 0.49-0.70; Δ=+0.25,+73.0%). Comparable trends were observed in RealClin, where image-augmented performance exceeded history-only performance by 23.4%‐57.0% across all 10 models, confirming that radiologist-selected 2D images enhance diagnostic performance across diverse clinical contexts and model architectures.

In RealClin, adding volumetric data to clinical history likewise improved performance over the history-only baseline in most models, with gains of up to +35.7% at fps=2 (eg, InternVL3-8B: mean 0.42, 95% CI 0.35-0.49 to mean 0.57, 95% CI 0.49-0.66) and up to +34.6% at fps=10 (eg, Gemini-2-Flash: mean 0.52, 95% CI 0.45-0.61 to mean 0.70, 95% CI 0.61-0.80). Two models showed no benefit from volumetric input compared with history alone: Llama-4-Scout-17B-16E and MedGemma-4B; both could only be evaluated at fps=2 due to context-window and GPU-memory constraints. However, when compared against the much simpler radiologist-selected 2D image baseline, volumetric input degraded performance in the majority of models, revealing a fundamental paradox between input completeness and diagnostic utility. At fps=2, a total of 8 of 10 models declined relative to the clinical history+images condition, with reductions ranging from −6.8% (Qwen2.5-VL-72B: mean 0.71, 95% CI 0.62-0.81 to mean 0.66, 95% CI 0.57-0.75) to −28.6% (Llama-4-Scout-17B-16E: mean 0.59, 95% CI 0.51, 0.69 to mean 0.42, 95% CI 0.35-0.50); only 2 smaller models (Qwen2.5-VL-7B and InternVL3-8B) showed marginal improvement (+2.4% and +2.1%, respectively). At fps=10, all 8 evaluable models declined relative to the 2D-image condition, with reductions ranging from −5.3% (Qwen2.5-VL-7B) to −31.4% (Gemini-2.5-Flash-Thinking). Comparison of the 2 volumetric sampling rates further demonstrated that providing more frames did not translate into better diagnostic performance: for 6 of the 8 models evaluable at both rates, fps=10 produced equivalent or worse results than fps=2. The most pronounced fps-dependent deterioration was observed for Gemini-2.5-Flash-Thinking (0.61 at fps=2 vs 0.54 at fps=10) and InternVL3-78B (0.68 vs 0.57), whereas Gemini-2-Flash (0.69 vs 0.70) and O3 (0.68 vs 0.72) were relatively stable. These findings indicate that current MLLMs not only fail to extract incremental diagnostic value from raw volumetric data beyond what is already conveyed by radiologist-selected key slices but also struggle to exploit additional temporal sampling, suggesting that the bottleneck lies in the models’ inability to identify and prioritize diagnostically salient slices rather than in the absence of volumetric information.

### Case-Level Stability and Score Distribution Analysis

**Figure 4.**
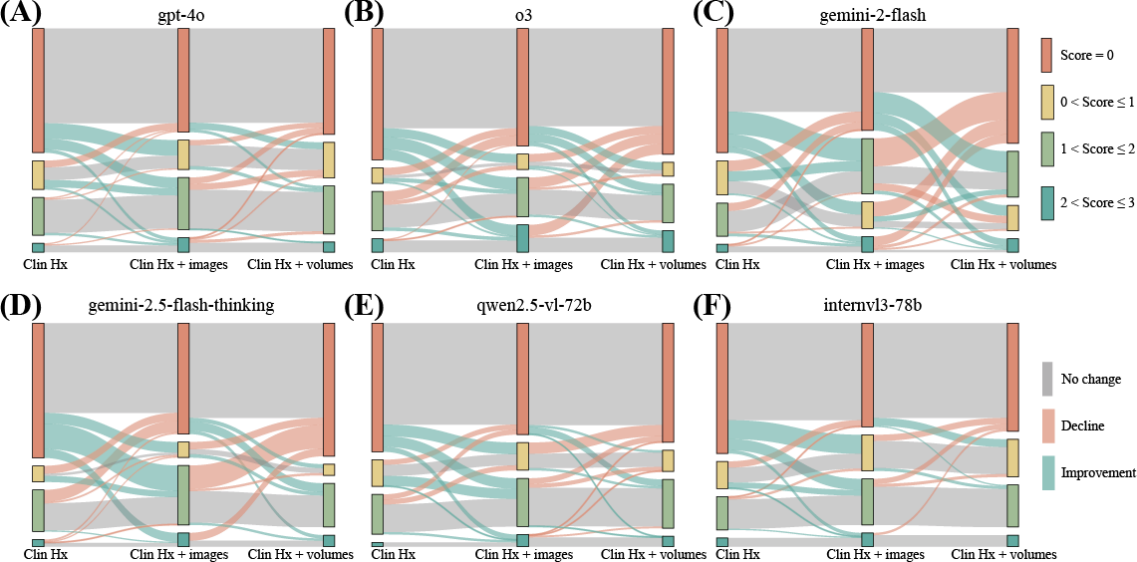
Performance transition analysis across input modalities in the RealClin dataset at fps=2. Sankey diagrams illustrating score transitions for 6 representative models, as input modalities progress from clinical history only (Clin Hx) to clinical history plus key images (Clin Hx+images) to clinical history plus volume data sampled at 2 fps (Clin Hx+volumes). (A) gpt-4o, (B) o3, (C) gemini-2-flash, (D) gemini-2.5-flash-thinking, (E) qwen2.5-vl-72b, and (F) internvl3-78b. Flow width represents the proportion of cases transitioning between score ranges: 0 (red), 0<score≤1 (yellow), 1<score≤2 (green), and 2<score≤3 (teal). Flow colors indicate per-case performance changes between adjacent input conditions: gray (no change), red (decline), and teal (improvement). Equivalent transitions for the fps=10 sampling condition are shown in Figure S2 in Multimedia Appendix 2.

Individual case trajectory analysis using Sankey plots (Figure 4 and Figure S2 and Table S5 in Multimedia Appendix 2) revealed distinct stability patterns across input formats and sampling rates, highlighting model-specific sensitivities to visual input format that complement the aggregate score analysis above. The transition from clinical history to image-enhanced input demonstrated universally beneficial effects across all 6 representative high-capacity models, with 20.0%‐27.8% of cases improving while deterioration was limited to 4.4%‐12.2%. Gemini-2-Flash achieved the most favorable transition profile (100/360, 27.8% improvement), while InternVL3-78B demonstrated the lowest deterioration rate (16/360, 4.4%). This case-level evidence confirms that expert-curated 2D images reliably enhance diagnostic performance with minimal risk across architectures.

The transition from 2D images to volumetric data revealed sampling-rate–dependent patterns of diagnostic volatility (Table S5 in Multimedia Appendix 2). At fps=2, a total of 5 of the 6 representative models maintained high consistency, with 68.9%‐86.1% of predictions unchanged (InternVL3-78B: 310/360, 86.1%, GPT-4o: 296/360, 82.2%, Qwen2.5-VL-72B: 296/360, 82.2%, O3: 254/360, 70.6%, and Gemini-2.5-Flash-Thinking: 248/360, 68.9%); however, this case-level stability did not guarantee score preservation, as Gemini-2.5-Flash-Thinking showed the largest absolute score deterioration at fps=2 (−0.19 points, −23.7% relative to its 2D performance) despite 68.9% of its individual predictions remaining unchanged. Gemini-2-Flash was a notable exception in stability, exhibiting extensive bidirectional reinterpretation (86/360, 23.9% improved vs 113/360, 31.4% declined, 161/360, 44.7% unchanged), although its overall score change (−0.13, −15.8%) was less severe than that of Gemini-2.5-Flash-Thinking. At fps=10 (Table S5 and Figure S2 in Multimedia Appendix 2), the patterns shifted markedly: GPT-4o exhibited the most pronounced “exchange phenomenon” (110/360, 30.6% improved but 129/360, 35.8% declined), resulting in wholesale reinterpretation of two-thirds of predictions rather than incremental refinement; Gemini-2.5-Flash-Thinking again showed the largest absolute score deterioration (−0.25 points, −31.4%; 35/360, 9.7% improved vs 80/360, 22.2% declined). Among the 6 models, Qwen2.5-VL-72B retained the high case-level stability under both sampling rates (296/360, 82.2% unchanged at fps=2 and 297/360, 82.5% unchanged at fps=10), suggesting an architecturally distinctive robustness to volumetric input format. Together, these case-level findings demonstrate that volumetric processing introduces substantial diagnostic variability without compensatory gains, and that for most models, additional temporal sampling at fps=10 amplifies rather than mitigates this variability, reinforcing the architectural limitations identified in the aggregate-score analysis.

### Cross-Dataset Generalization: RadEdu vs RealClin

Cross-dataset evaluation between RadEdu (English, publicly available teaching cases) and RealClin (Chinese, routine hospital studies drawn from an internal hospital database that was not publicly released, although exposure of similar regional clinical data during open-source model pretraining cannot be excluded) revealed fundamental differences in model generalization that illuminate the complex interplay between training data exposure, language alignment, and architectural design (Figure 3 and Table S1 in Multimedia Appendix 2). Proprietary models consistently peaked in RadEdu and declined substantially in RealClin. O3 showed the most pronounced deterioration, from 1.14 (95% CI 1.02-1.28) to 0.79 (95% CI 0.69-0.90) in image-enhanced performance (Δ=−0.35); Gemini-2.5-Flash-Thinking and GPT-4o showed similar declines. This consistent deterioration is consistent with several nonexclusive factors, including potential overlap of RadEdu cases with web-accessible educational content used in pretraining, cross-linguistic generalization effects, and differences in clinical complexity, multimorbidity, atypical or borderline findings, and case realism between the 2 datasets. Conversely, large open-source models exhibited contrasting performance patterns, with Chinese-centric architectures demonstrating particularly pronounced improvements in RealClin. InternVL3-78B increased from 0.48 (95% CI 0.39-0.58) in RadEdu to 0.75 (95% CI 0.66-0.85) in RealClin (Δ=+0.27), while Qwen2.5-VL-72B improved from 0.59 (95% CI 0.49-0.70) to 0.71 (95% CI 0.62-0.81; Δ=+0.12). Notably, their smaller variants (7B/8B) displayed even greater relative improvements, with scores approximately doubling from RadEdu to RealClin. These open-source models therefore showed a cross-dataset performance trajectory in the opposite direction to that of the proprietary models. This directional contrast was further reflected in the dataset-dependent utility of static 2D images. Proprietary models showed significantly greater improvements in RadEdu (median Δ=+0.46) compared to RealClin (median Δ=+0.25), whereas large open-source models exhibited more consistent gains (median Δ=+0.17 vs +0.20). These findings illustrate how benchmark source can substantially alter model rankings and underscore the importance of reporting real-world generalization alongside performance on publicly available educational datasets.

### Cross-Linguistic Control Experiment

To rule out language as a confounder in the cross-dataset comparison, we re-evaluated all 10 models in RealClin after translating every clinical history from Chinese into English (Table S6 in Multimedia Appendix 2). Translation produced a numerical decrease in mean diagnostic score for all 10 of 10 models, with the decrease reaching statistical significance for 9 of 10 (range Δ=−0.03 to −0.19; BH-adjusted *P* from <.001 to .38). The decline was observed across model categories, including English-centric proprietary systems that would be expected to favor English input (O3: mean 0.59, 95% CI 0.49-0.68 vs mean 0.40, 95% CI 0.32-0.48; Δ=−0.19; *P*<.001; GPT-4o: Δ=−0.10; *P*=.03), Chinese-centric open-source models (Qwen2.5-VL-72B: Δ=−0.18; *P*<.001; InternVL3-78B: Δ=−0.16; *P*<.001), and the medical-English-fine-tuned MedGemma-4B (Δ=−0.19, *P*<.001). Only Llama-4-Scout-17B-16E showed a nonsignificant trend (Δ=−0.03; *P*=.38). The observation that even predominantly English-trained models, including a medical-English-fine-tuned model, performed worse on translated English than on original Chinese refutes the hypothesis that Chinese-language input imposes a performance penalty in RealClin and suggests that the lower RealClin scores observed in the main analysis reflect genuine differences in clinical content and case realism rather than a language artifact. The most plausible explanation for this uniform decline is information loss during machine translation. Domain-specific terminology, idiomatic expressions, and contextual cues in the original Chinese histories can be diluted or altered on conversion to English. Because machine translation can itself omit or alter information, the translated condition is not a clean language-only manipulation. The translated scores should therefore be read as a conservative bound rather than a faithful language-matched comparison.

### Impact of Disease Rarity on Cross-Dataset Performance

**Figure 5.**
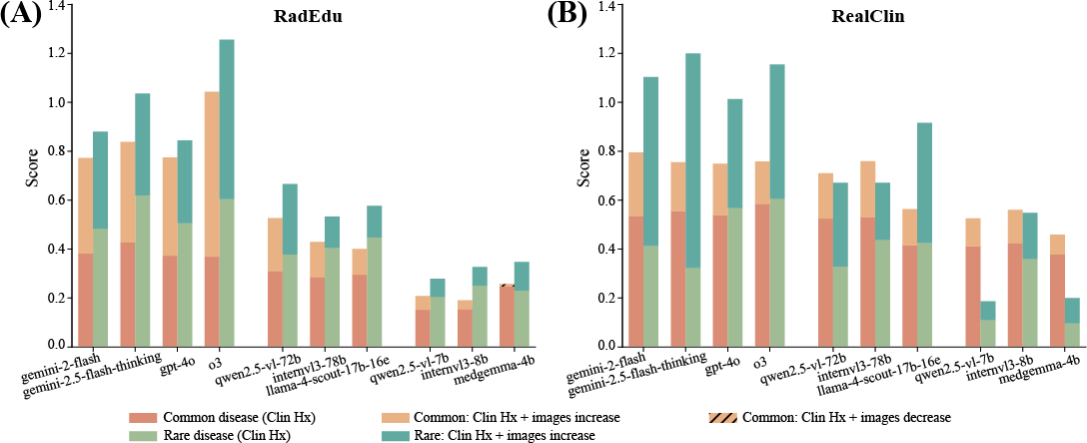
Model performance stratified by disease rarity. Performance comparison between common and rare disease cases across clinical history only (Clin Hx) and clinical history plus images (Clin Hx+images) conditions. (A) RadEdu dataset showing baseline performance for common diseases (orange bars) and rare diseases (green bars) with clinical history alone, and corresponding performance changes when images are added (lighter colored bars indicate increase and hatched bars indicate decrease). (B) RealClin dataset with identical methodology and color coding.

The marked difference in rare disease frequency between datasets (RadEdu 164/360, 45.6% vs RealClin 31/360, 8.6%; *P*<.001) enabled a stratified analysis of how disease prevalence interacts with model performance under different input modalities (Figure 5 and Table S7 in Multimedia Appendix 2).

For the purposes of this analysis, we define the rare-disease premium for a given model as the arithmetic difference between that model’s mean diagnostic score on rare-disease cases and its mean diagnostic score on common-disease cases, computed within the same dataset and under the same input condition; a positive premium denotes higher performance on rare than on common diseases, and a “reversal” of the premium denotes a change in the sign of this difference between datasets or input conditions.

In RadEdu, 9 of 10 models demonstrated a “rare-disease premium” under the clinical history only condition, with rare disease scores exceeding common disease scores (eg, O3: 0.60 vs 0.37; Gemini-2.5-Flash-Thinking: 0.62 vs 0.43; Qwen2.5-VL-7B: 0.20 vs 0.15); MedGemma-4B was the only exception (common: 0.26 vs rare: 0.23). Adding radiologist-selected key images preserved this premium in all 10 of 10 models, with the largest absolute gaps observed for proprietary reasoning models (O3 rare: 1.26 vs common: 1.04; Gemini-2.5-Flash-Thinking rare: 1.04 vs common: 0.84).

In RealClin, this premium reversed for the majority of models. Under the clinical history only condition, common disease scores exceeded rare disease scores in 7 of 10 models (eg, Gemini-2-Flash: 0.53 vs 0.41; Gemini-2.5-Flash-Thinking: 0.56 vs 0.32), while 3 models showed comparable or slightly higher rare disease performance (GPT-4o: 0.54 vs 0.57; O3: 0.58 vs 0.61; Llama-4-Scout-17B-16E: 0.42 vs 0.43). Identifying rare clinical entities from text alone was therefore more difficult than identifying common ones for most models on this dataset, in contrast to the rare-disease premium observed on the public teaching corpus.

The introduction of key images produced a striking divergence between model categories in RealClin. In total, 5 models ended with rare disease scores exceeding common disease scores (Gemini-2-Flash: 1.10 vs 0.80; Gemini-2.5-Flash-Thinking: 1.20 vs 0.76; GPT-4o: 1.01 vs 0.75; O3: 1.15 vs 0.76; Llama-4-Scout-17B-16E: 0.92 vs 0.56), while the remaining 5 models retained higher common than rare disease scores (Qwen2.5-VL-72B: 0.71 vs 0.67; InternVL3-78B: 0.76 vs 0.67; Qwen2.5-VL-7B: 0.53 vs 0.19; InternVL3-8B: 0.56 vs 0.55; MedGemma-4B: 0.46 vs 0.20). The 2 Chinese-centric large open-source models, in particular, retained markedly stronger performance on common diseases under multimodal input, whereas the 4 proprietary models and Llama-4-Scout-17B-16E recovered a “rare-disease premium.” These divergent multimodal patterns indicate that the rare-disease premium of public teaching corpora is not uniformly reproduced in real-world Chinese clinical practice, and that current open-source Chinese-centric MLLMs preserve a different disease-prevalence behavior on real clinical cases than proprietary systems.

### Comparative Performance of Reasoning-Optimized Versus Standard Proprietary Models

To evaluate the clinical value of chain-of-thought reasoning capabilities, we compared 2 pairs of commercially available MLLMs that differ primarily in reasoning optimization: Gemini-2-Flash vs Gemini-2.5-Flash-Thinking and GPT-4o vs O3 (Table S1 in Multimedia Appendix 2).

In RadEdu, reasoning variants demonstrated consistent superiority across all evaluation conditions. For history-only input, Gemini-2.5-Flash-Thinking exceeded Gemini-2-Flash by 0.09 absolute scores (0.52 vs 0.43), while O3 exceeded GPT-4o by 0.05 (0.48 vs 0.43). The advantage widened substantially with key images: +0.11 for the Gemini pair (0.93 vs 0.82) and +0.33 for the GPT/O3 pair (1.14 vs 0.81). Notably, reasoning models achieved greater performance gains from image incorporation in both pairs (Gemini +0.41 vs +0.39; O3 +0.66 vs +0.37).

However, this pattern reversed in RealClin. Performance gaps narrowed to ≤0.05 for history-only input (0.54 vs 0.52 for Gemini; 0.59 vs 0.54 for GPT/O3). With key images, the Gemini ranking inverted (reasoning 0.79, baseline 0.82; −0.03), while O3 retained only a marginal lead over GPT-4o (0.79 vs 0.77; +0.02). The introduction of volumetric data further degraded reasoning model performance at both sampling rates. At fps=2, the Gemini inversion persisted (0.61 vs 0.69; −0.08), and the GPT or O3 pair essentially equalized (0.68 vs 0.69; −0.01). At fps=10, the Gemini inversion deepened further (0.54 vs 0.70; −0.16), while the GPT or O3 pair showed a slight advantage for O3 (0.72 vs 0.69; +0.03). Overall, reasoning-optimized variants achieved substantial diagnostic performance improvements on the curated RadEdu benchmark but showed minimal or negative improvements on the real-world RealClin benchmark, particularly after adding volumetric data.

## Discussion

### Principal Findings

This study presents RadM-Bench, a bilingual radiology benchmark that pairs publicly indexed English-language teaching cases (RadEdu) with Chinese routine-clinical cases (RealClin) drawn from a nonpublic hospital archive, and that evaluates each case under clinical-history-only, expert-curated 2D image, and volumetric (fps=2 and fps=10) input conditions. We used a hierarchical diagnostic scoring system reflecting the graded nature of radiological reasoning, rewarding early correct diagnoses while still crediting partially correct answers that may inform patient management decisions. Under this framework, 10 state-of-the-art MLLMs scored below 1.5 on a 0‐3 scale on both datasets, and 3 quantitative patterns recurred across model families. First, adding radiologist-curated 2D key images to clinical history improved performance in 10 of 10 models, with relative gains of +19.8% to +139.2%. Second, raw volumetric input did not match this 2D-image baseline: at fps=10, all 8 of 8 evaluable models scored lower with volumetric input than with the 2D-image baseline (range −5.3% to −31.4%), and at fps=2, a total of 8 of 10 models declined relative to the 2D baseline (range −6.8% to −28.6%), with Qwen2.5-VL-7B (+2.4%) and InternVL3-8B (+2.1%) as the only exceptions; MedGemma-4B and Llama-4-Scout-17B-16E could only be evaluated at fps=2 owing to context-window and GPU-memory constraints. Third, cross-dataset transfer diverged systematically by model category: the 4 proprietary models declined from RadEdu to RealClin (eg, O3 with images: 1.14 to 0.79), whereas the 2 Chinese-centric large open-source models improved in RealClin (eg, InternVL3-78B: 0.48 to 0.75), and the rare-disease premium present in 9 of 10 models in RadEdu disappeared or reversed in RealClin, where common-disease scores exceeded rare-disease scores in 7 of 10 models.

### Comparison to Prior Work

RadM-Bench occupies a region of the medical-multimodal benchmark design space that is complementary to, rather than overlapping with, 2 recently described benchmarks. RadFM and its associated RadBench introduced large-scale evaluation of medical foundation models on combined 2D and 3D radiologic data [30], but the benchmark and its underlying training corpus are constructed exclusively in English and do not contrast routine clinical cases against publicly indexed teaching content. PadChest-GR provides bilingual Spanish-English grounded reporting on 4555 frontal chest X-ray studies [31] but is restricted to a single anatomic region and a single 2D modality. RadM-Bench extends this landscape by simultaneously providing (1) a Chinese-English bilingual axis sourced from a nonpublic clinical archive paired with publicly indexed teaching cases, enabling the provenance contrast central to our cross-dataset analysis; (2) volumetric CT and MRI evaluated alongside expert-curated 2D key images on the same patients, enabling the within-case 2D versus 3D quantitative contrast at 2 controlled frame-sampling rates; and (3) cross-subspecialty coverage balanced at 40 cases per subspecialty across 9 radiological subspecialties. The empirical findings reported here, particularly the cross-linguistic control experiment, the cross-dataset proprietary- versus open-source divergence, and the disappearance of the rare-disease premium in RealClin, depend on this combined design and would not have been recoverable in RadFM or RadBench or PadChest-GR individually.

The performance degradation with volumetric data reveals a fundamental paradox: while comprehensive imaging analysis is essential in clinical practice, current general-purpose MLLMs do not synthesize raw volumetric input into diagnostic value comparable to that obtained from a small set of radiologist-curated 2D key images. This highlights a fundamental gap between MLLM benchmarking settings and clinical radiology realities. Key 2D images consistently boost model performance, creating false impressions of deployment readiness [4], whereas full volumetric data that radiologists routinely interpret introduces diagnostic volatility. RadM-Bench’s dual-format design was crucial for exposing this limitation; evaluations restricted to 2D images would have overestimated clinical utility. The diagnostic instability observed under volumetric input, particularly the case-level prediction reinterpretations seen in models such as GPT-4o, is consistent with current MLLMs being unable to identify and prioritize diagnostically salient slices on their own; rather than the absence of volumetric information, the bottleneck appears to be slice-level salience selection within long frame sequences.

The contrasting generalization patterns between proprietary and open-source models across datasets provide critical insights into MLLMs’ preparedness for clinical deployment. Proprietary models’ consistent decline from educational to clinical datasets is in line with prior exposure to web-accessible educational content during pretraining, with limitations in cross-linguistic medical reasoning, or with both. Three nonexclusive factors explain these contrasting transfer patterns: (1) potential training-set exposure—RadEdu teaching cases have been openly indexed online for many years and may have been encountered by general-purpose MLLMs during pretraining, although we did not perform direct contamination or memorization testing, and a quantitative attribution to data exposure cannot be established from our data alone; (2) language alignment—Chinese-centric open-source models are explicitly trained on Chinese text, providing vocabulary and syntax matching with RealClin’s clinical histories; and (3) clinical complexity—RealClin’s routine imaging contains multimorbidity and borderline findings differing from RadEdu’s textbook-style exemplars, so that architectures relying heavily on prototypical cues may struggle while models trained with diverse, noise-tolerant objectives maintain or improve performance. The cross-linguistic control experiment additionally rules out a Chinese-language input penalty as the dominant explanation for the proprietary-model decline. This control carries one caveat. The translation step can itself introduce semantic drift. Domain-specific terminology and contextual cues in the original Chinese histories may be diluted or altered on conversion to English. This is consistent with reports of information loss in machine-translated clinical text [32]. The lower scores on translated English therefore partly reflect translation loss rather than language preference alone. The experiment establishes the absence of a Chinese-input penalty, not a perfectly language-matched comparison. The observation that benchmark provenance can decisively reshape model rankings has direct implications for clinical AI evaluation: rankings derived from public educational datasets alone may not predict rankings on routine-clinical data drawn from a different linguistic and institutional context.

The contrast between rare- and common-disease performance differed sharply between the 2 datasets. In RadEdu, 9 of 10 models showed a positive rare-disease premium under the clinical-history–only condition, consistent with the educational dataset’s enrichment of classic, well-documented rare entities sourced from the US NORD catalog [18]. This benchmark therefore rewards recognition of textbook-stereotyped textual and visual cues that occur infrequently in daily clinical practice. In RealClin, this premium disappeared or reversed: under history-only input, common-disease scores exceeded rare-disease scores in 7 of 10 models. Three nonexclusive factors are consistent with this reversal: (1) the spectrum of rare diseases encountered in Chinese clinical practice differs from NORD-derived rare-disease distribution that dominates English-language teaching corpora; (2) Chinese medical terminology is underrepresented in the predominantly English-language pretraining data of most proprietary models; and (3) atypical imaging features in routine clinical workflow place greater demands on visual analysis than the more prototypical presentations curated for teaching. With the addition of 2D key images, the 4 proprietary models and Llama-4-Scout-17B-16E recovered a positive rare-disease premium in RealClin, whereas the 2 Chinese-centric large open-source models retained higher performance on common diseases—a divergence suggesting that architecture and scale can partially compensate for cross-linguistic and cross-prevalence shifts when high-quality visual evidence is available.

In RealClin, the 2 reasoning-augmented proprietary models (O3 and Gemini-2.5-Flash-Thinking) did not show a volumetric-input advantage that might have been expected from their explicit step-by-step reasoning capability. At fps=10, both reasoning models scored lower with volumetric input than with the 2D-image baseline, with Gemini-2.5-Flash-Thinking declining from 0.79 to 0.54 (a 31.4% reduction). In the present setting, extended chain-of-thought reasoning over long frame sequences did not translate into improved diagnostic ranking on routine clinical cases relative to a much shorter, expert-curated 2D presentation—consistent with the broader pattern that, when high-quality visual evidence is available, the bottleneck for current MLLMs is salience selection over long frame sequences rather than the depth of textual reasoning.

### Limitations

Several limitations of this study should be acknowledged. First, our volumetric pipeline differs from the radiologist’s interactive picture archiving and communication system workflow. Volumetric studies were presented to MLLMs as linear frame sequences (fps=2 or fps=10) extracted from H.264-encoded MP4 files exported with clinician-selected window and level settings, whereas radiologists scroll dynamically, adjust window and level on the fly, and reformat in multiple planes during clinical reading. To mitigate this gap as far as is currently possible, the same expert-curated MP4 source was used for every model, the window and level for each case were chosen in advance by a board-certified radiologist, and 2 standardized temporal sampling rates (fps=2 and fps=10) were evaluated to bracket the trade-off between token economy and temporal completeness. Two sources of information loss are nevertheless inherent: lossy H.264 compression and the conversion from native 12‐16 bit DICOM to 8-bit RGB (red, green, blue). The observed 2D versus 3D performance gap should therefore be interpreted as reflecting each model’s ability to extract diagnostic information from sequential, clinician-windowed frame presentations rather than from a fully interactive volumetric review at native bit depth, and this gap may narrow once MLLMs that support DICOM-native input and interactive viewing become available. Second, the benchmark is limited in scale (n=720), and the RealClin component was drawn from a single Chinese academic tertiary hospital. We mitigated this by stratifying both subsets across 9 radiological subspecialties (40 cases per subspecialty per dataset) and by pairing every Chinese clinical history with its English translation for the cross-linguistic control analysis, but findings in RealClin may still carry institution-specific imaging conventions, reporting templates, and local disease prevalence patterns. Cross-dataset transfer results should therefore be retested on additional Chinese centers and on routine clinical cohorts from other countries before being treated as broadly representative. Third, the linguistic coverage of RadM-Bench is restricted to Chinese and English. The cross-linguistic control experiment described in the Results section addresses the specific concern that Chinese-language input penalizes model performance in RealClin, but it does not speak to clinical settings operating in Spanish, Arabic, Hindi, or other widely used clinical languages, where vocabulary coverage in current general-purpose MLLMs may differ substantially. The cross-linguistic findings reported here should therefore be regarded as a Chinese-English-specific signal, and future benchmarks should extend cross-linguistic evaluation to additional languages and writing systems. Fourth, the 10 models evaluated here represent a snapshot of the proprietary, large open-source, and small open-source MLLM landscape as of early 2026 and do not exhaust emerging architectures, including models with native medical-imaging adapters, agentic radiology pipelines, and models that support DICOM-native input. The benchmark construction and evaluation pipeline were designed to be model-agnostic, and the RealClin dataset and evaluation code have been released to allow newer models to be assessed against the same protocol; nevertheless, the specific quantitative comparisons reported here are time-bounded and should be rerun as the model landscape evolves. Fifth, evaluation was limited to ranked differential diagnoses scored under a 4-tier diagnostic-quality rubric. We did not formally quantify hallucinated findings, misleading explanatory text, or inappropriate downstream recommendations that may co-occur with correct diagnoses, and we did not assess longitudinal effects on patient management or outcomes. The present scoring framework therefore answers whether the correct diagnosis appears at a clinically actionable rank but does not answer whether the rest of the model’s output is safe to expose to a treating clinician.

Deployment-oriented studies should extend the evaluation protocol to include structured error-mode taxonomies (hallucinations, omissions, and recommendation errors) and prospective end points linked to clinical decision-making.

### Future Directions

Several directions follow directly from the limitations identified above and from the empirical patterns observed in this study. First, MLLM architectures that natively accept DICOM input, support interactive viewing actions (scrolling, window and level adjustment, and multiplanar reformatting), and preserve the original 12‐ to 16-bit dynamic range are needed before volumetric performance can be assessed under conditions that match the radiologist’s actual diagnostic workflow. Second, multicenter and multinational extensions of RealClin would help to disentangle genuine cross-dataset transfer effects from institution-specific imaging and reporting conventions; analogous extensions to additional clinical languages would test whether the cross-linguistic patterns observed here generalize beyond the Chinese-English contrast, and harmonization of cross-lingual disease terminology and rare-disease taxonomies would support comparable reporting across regions. Third, the evaluation protocol should be extended beyond diagnostic ranking to include structured assessment of hallucinations, omissions, recommendation appropriateness, downstream effects on clinical decision-making, and disease rarity–stratified reporting so that strong performance on rare entities does not mask weakness on common ones (or vice versa), ideally in prospective deployment studies with clinician-in-the-loop comparators. Fourth, because mean performance of all 10 models remained below 1.5 on the 0‐3 scale, targeted methodological work on volumetric reasoning—including architectures that efficiently model long-range spatial-temporal dependencies and training objectives that enforce case-level rather than frame-level consistency—will be required to close the gap to clinically viable diagnostic performance. Fifth, the absence of a uniform volumetric or reasoning-model advantage in RealClin suggests that defaulting to the most computationally expensive model is not warranted in routine clinical settings where high-quality 2D evidence is available; cost-aware, adaptive deployment strategies that route cases to lighter-weight models when 2D evidence is unambiguous and reserve reasoning-augmented or larger models for ambiguous or visually atypical cases should be evaluated prospectively against fixed-model baselines in real workflows.

### Conclusions

Our study characterizes the performance of current MLLMs in radiological diagnosis through comprehensive evaluation on our developed RadM-Bench dataset across input modalities, clinical settings, disease rarity, and linguistic contexts. While multimodal inputs improved performance over clinical history alone, performance gaps remain in volumetric data processing and cross-context generalization, with mean diagnostic performance across the 10 evaluated models remaining below clinically actionable levels on both datasets.

## Supplementary material

10.2196/92183 Multimedia Appendix 1 Prompts, per-model frame sampling, tokenization, and token-budget verification.

10.2196/92183 Multimedia Appendix 2 Supplementary figures and tables reporting detailed model performance.

## Abbreviations

BH: Benjamini-Hochberg
CT: computed tomography
DICOM: Digital Imaging and Communications in Medicine
FPS: frames per second
GPU: graphics processing unit
MLLM: multimodal large language model
MR: magnetic resonance
MRI: magnetic resonance imaging
NORD: National Organization for Rare Disorders
