# Supplementary material for: A Bilingual Benchmark for Evaluating Diagnostic Performance of Multimodal Large Language Models in Radiology (RadM-Bench): Evaluation Development and Validation
Source: J Med Internet Res. 2026 Aug 7;28:e92183. doi: 10.2196/92183 (PMC13450880; doi:10.2196/92183)
Supplement: Supplementary file 1 [file jmir-v28-e92183-s001.docx]

**Prompts**

The prompts used in this study:

prompt_en = """

Based on the provided Clinical History and images/videos, please provide 5 possible diagnoses in order of likelihood.

Clinical History: {clinical_history}

Respond strictly in the following JSON format without any additional words or explanations:

{{

"diagnoses": [

"Diagnosis 1 (most likely)",

"Diagnosis 2",

"Diagnosis 3",

"Diagnosis 4",

"Diagnosis 5 (least likely)"

]

}}

"""

prompt_zh = """

根据提供的临床病史和图像/视频，请按照可能性从高到低提供5个可能的诊断。

临床病史: {clinical_history}

请严格按照以下JSON格式回答，不要添加任何额外的文字或解释：

{{

"diagnoses": [

"诊断1中文（最可能）",

"诊断2中文",

"诊断3中文",

"诊断4中文",

"诊断5中文（最不可能）"

]

}}

"""

sys_prompt = f"""

"You are a helpful AI assistant that provides concise medical diagnoses based on clinical history and images/videos. You must output only the 5 most likely diagnoses, strictly in JSON format as specified, without any additional details or explanations."

"""

**Per-model frame sampling, tokenization, and token-budget verification**

**Frame sampling rate**

Volumetric studies were evaluated under two temporal sampling conditions:

(a) fps=10 (full frame rate): all original frames from each MP4 clip were supplied to the model, either as native video input or as sequential frame stacks. For the median case (163 original frames), all 163 frames were provided; for the longest case (1,024 frames), all 1,024 frames were provided.

(b) fps=2 (subsampled frame rate): frames were uniformly subsampled from the original 10-fps clips. The number of sampled frames N per clip was calculated as N = floor_even( (T_total / f_video) x f_sample ), where T_total is the total number of original frames, f_video = 10 fps, and f_sample = 2 fps; floor_even denotes rounding down to the nearest even integer. For the median case this yields N = 32 (effective rate 1.96 fps); for the longest case N = 204 (effective rate 1.99 fps). Sampled frames were selected at uniformly spaced indices across the full clip duration.

Frame-rate control was implemented through each model's native interface:

- Qwen2.5-VL (72B and 7B): the qwen-vl-utils library's sampling rate was explicitly set to 10 fps and 2 fps for the two conditions.

- Gemini-2.0-Flash and Gemini-2.5-Flash-Thinking: complete MP4 files were uploaded with VideoMetadata(fps=10) or VideoMetadata(fps=2) set explicitly via the Gemini API.

- InternVL3 (78B and 8B), GPT-4o, O3, MedGemma-4B, and LLaMA-4-17B-16E: because these models do not accept native video input via the API or open-source interface, frames were pre-extracted as JPEGs and supplied as sequential image stacks at each target rate.

- MedGemma-4B and LLaMA-4-17B-16E: at fps=10 these models could not be evaluated owing to context-window and GPU-memory constraints (see (2) below); results for these two models are therefore reported only at fps=2. All the other eight models were successfully evaluated at both fps=2 and fps=10.

**Token-limit handling**

Vision-language models convert each input frame into a sequence of visual tokens whose total count, together with text tokens, must fit within the model's context window. We verified per-case token utilization at both frame rates for every model.

Qwen2.5-VL (nominal context window 32K, extensible to 128K via YaRN): images are divided into 28 x 28 pixel patches, and a built-in dynamic resolution mechanism reduces per-frame spatial resolution when the total visual-token count approaches the context limit. At fps=2, frames were resized to 504 x 504 pixels, yielding 324 tokens per frame (median case 10,368 tokens; maximum 66,096 tokens). At fps=10, the dynamic resolution mechanism progressively reduced per-frame resolution (down to the minimum 28 x 28 pixel patch grid when necessary) so that all cases fit within the extended context window. One case (1,024 frames) produced a position-index warning but still returned valid output, attributable to Qwen2.5-VL's M-RoPE with NTK-aware scaling, which permits graceful handling of inputs marginally exceeding the nominal window.

InternVL3 (nominal context window 32K tokens, extensible for long multimodal inputs via Variable Visual Position Encoding, V2PE): unlike conventional position encoding where every token increments the position index by 1, V2PE assigns a fractional position increment delta (delta < 1) to visual tokens, so that many visual tokens share a small range of position IDs. InternVL3 uses an InternViT encoder with 14 x 14 pixel patches followed by pixel-shuffle downsampling, yielding 256 visual tokens per 448 x 448 frame. Raw visual token counts at fps=2 are 8,192 (median) and 52,224 (maximum); at fps=10 they are 41,728 (median) and 262,144 (maximum). With V2PE applied at a stride of 64 (giving delta = 1/64), the effective position indices for all cases reindices for all cases remained well within the 32K window (e.g., the maximum case at fps=10 corresponded to approximately [262144/64=4096] visual position IDs). All cases completed successfully without truncation at either frame rate. This mechanism is described in detail in the V2PE paper (Ge et al., arXiv:2412.09616) and the InternVL3 technical report (Zhou et al., arXiv:2504.10479).

Gemini-2.0-Flash and Gemini-2.5-Flash-Thinking (context window 1,048,576 tokens): per Google's API documentation, each video frame consumes approximately 258 tokens. At fps=2, median ~8,256 tokens; maximum ~52,632 tokens. At fps=10, median ~42,054 tokens; maximum ~264,192 tokens. All values represent less than 26% of the 1M-token context window; no truncation or error occurred at either frame rate.

GPT-4o and O3 (context window 128K tokens): the internal tokenizer is proprietary, but per OpenAI's documentation each image consumes approximately 170 to 1,105 tokens depending on resolution and the "detail" parameter; we used the default "auto" setting. All cases completed without context-length errors at both frame rates.

MedGemma-4B (context window 128K tokens): despite a nominal context size comparable to Qwen2.5-VL (extended) and GPT-4o, MedGemma-4B inherits Gemma 3's hybrid sliding-window / periodic-global attention architecture with pre-allocated fixed-size KV-cache buffers. When visual-token counts approached the 128K boundary at fps=10, this produced a context-length error rather than graceful degradation, preventing valid output on most cases. At fps=2, all cases fit within the fixed buffer and completed successfully. Results are therefore reported only for the fps=2 condition.

LLaMA-4-17B-16E: at fps=10 the model exceeded available GPU memory on our 8 x H20 (141 GB each) cluster for most cases; at fps=2 memory requirements were reduced to a feasible range and all cases completed. Results are therefore reported only for the fps=2 condition.
