# Supplementary material for: A Bilingual Benchmark for Evaluating Diagnostic Performance of Multimodal Large Language Models in Radiology (RadM-Bench): Evaluation Development and Validation
Source: J Med Internet Res. 2026 Aug 7;28:e92183. doi: 10.2196/92183 (PMC13450880; doi:10.2196/92183)
Supplement: Supplementary file 2 [file jmir-v28-e92183-s002.docx]

**Figure S1. Top-1, Top-3 and Top-5 accuracy of models across input modalities and datasets. (A, C, E) Top-1, Top-3 and Top-5 accuracy of 10 multimodal large language models in RadEdu cases under two input conditions: clinical history only (Clin Hx, red), and clinical history plus key images (Clin Hx + Images, yellow). (B, D, F) Top-1, Top-3 and Top-5 accuracy in RealClin cases under four input conditions: clinical history only (Clin Hx, red), clinical history plus key images (Clin Hx + Images, yellow), clinical history plus volumetric data sampled at 2 fps (Clin Hx + Volumes 2fps, light teal), and clinical history plus volumetric data at 10 fps (Clin Hx + Volumes 10fps, dark teal). LlaMA-4-Scout-17B-16E and MedGemma-4B were evaluated only at fps=2 owing to context-window and GPU-memory constraints. Error bars represent 95% confidence intervals.**

**Figure S2. Performance transition analysis across input modalities in RealClin dataset at fps=10.** Sankey diagrams illustrating score transitions for six representative models as input modalities progress from clinical history only (Clin Hx) to clinical history plus key images (Clin Hx + Images) to clinical history plus volume data sampled at 10 fps (Clin Hx + Volumes). **(A)** GPT-4o, **(B)** O3, **(C)** Gemini-2-Flash, **(D)** Gemini-2.5-Flash-Thinking, **(E)** Qwen2.5-VL-72b, and **(F)** InternVL3-78b. Flow width represents the proportion of cases transitioning between score ranges: 0 (red), 0 < Score ≤ 1 (yellow), 1 < Score ≤ 2 (green), and 2 < Score ≤ 3 (teal). Flow colors indicate per-case performance changes between adjacent input conditions: gray (no change), red (decline), and teal (improvement).

**Table S1. Model performance across input modalities and datasets**

| **Models** | **RadEdu** | | |  | **RealClin** | | | | | |  |
| --- | --- | --- | --- | --- | --- | --- | --- | --- | --- | --- | --- |
|  | **Clin Hx** | **Clin Hx + Images** | **Δa** |  | **Clin Hx** | **Clin Hx + Images** | **Clin Hx + Volumes (2fps)** | **Clin Hx + Volumes (10fps)** | **Δa** | **Δb** | **Δc** |
| **Proprietary MLLMs** | | | | | | | | | | |  |
| gemini-2-flash | 0.43 [0.35, 0.53] | 0.82 [0.71, 0.94] | 0.39 (+91.3%) |  | 0.52 [0.45, 0.61] | **0.82 [0.72, 0.92]** | **0.69 [0.60, 0.79]** | 0.70 [0.61, 0.80] | 0.30 (+57.0%) | -0.13 (-15.8%) | -0.12 (-14.7%) |
| gemini-2.5-flash-thinking | **0.52 [0.43, 0.62]** | 0.93 [0.81, 1.04] | 0.41 (+80.2%) |  | 0.54 [0.45, 0.63] | 0.79 [0.70, 0.90] | 0.61 [0.51, 0.70] | 0.54 [0.45, 0.64] | 0.26 (+48.2%) | -0.19 (-23.7%) | -0.25 (-31.4%) |
| gpt-4o | 0.43 [0.35, 0.53] | 0.81 [0.70, 0.92] | 0.37 (+85.8%) |  | 0.54 [0.46, 0.63] | 0.77 [0.68, 0.87] | **0.69 [0.60, 0.79]** | 0.69 [0.60, 0.78] | 0.23 (+42.6%) | -0.08 (-10.2%) | -0.08 (-10.8%) |
| o3 | 0.48 [0.39, 0.59] | **1.14 [1.02, 1.28]** | 0.66 (+139.2%) |  | **0.59 [0.49, 0.68]** | 0.79 [0.69, 0.90] | 0.68 [0.58, 0.79] | **0.72 [0.62, 0.82]** | 0.21 (+35.1%) | -0.11 (-13.8%) | -0.07 (-8.7%) |
| **Large open-source MLLMs** | | | | | | | | | | |  |
| qwen2.5-vl-72b | 0.34 [0.27, 0.43] | **0.59 [0.49, 0.70]** | 0.25 (+73.0%) |  | 0.51 [0.43, 0.59] | 0.71 [0.62, 0.81] | 0.66 [0.57, 0.75] | **0.62 [0.53, 0.71]** | 0.20 (+39.2%) | -0.05 (-6.8%) | -0.09 (-12.2%) |
| internvl3-78b | 0.34 [0.27, 0.42] | 0.48 [0.39, 0.58] | 0.14 (+40.0%) |  | **0.52 [0.44, 0.62]** | **0.75 [0.66, 0.85]** | **0.68 [0.59, 0.78]** | 0.57 [0.48, 0.65] | 0.23 (+43.8%) | -0.07 (-9.5%) | -0.18 (-24.3%) |
| llama-4-scout-17b-16e | **0.36 [0.29, 0.47]** | 0.48 [0.39, 0.59] | 0.12 (+32.0%) |  | 0.42 [0.35, 0.50] | 0.59 [0.51, 0.69] | 0.42 [0.35, 0.50] | -- | 0.18 (+42.5%) | -0.17 (-28.6%) | -- |
| **Small open-source MLLMs** | | | | | | | | | | |  |
| qwen2.5-vl-7b | 0.18 [0.12, 0.24] | 0.24 [0.18, 0.32] | 0.06 (+36.6%) |  | 0.39 [0.32, 0.47] | 0.50 [0.42, 0.58] | 0.51 [0.43, 0.59] | 0.47 [0.40, 0.55] | 0.11 (+28.5%) | 0.01 (+2.4%) | -0.03 (-5.3%) |
| internvl3-8b | 0.20 [0.14, 0.26] | 0.25 [0.19, 0.33] | 0.05 (+27.1%) |  | **0.42 [0.35, 0.49]** | **0.56 [0.48, 0.64]** | **0.57 [0.49, 0.66]** | **0.52 [0.44, 0.61]** | 0.14 (+33.7%) | 0.01 (+2.1%) | -0.04 (-6.7%) |
| medgemma-4b | **0.24 [0.19, 0.31]** | **0.29 [0.22, 0.38]** | 0.05 (+19.8%) |  | 0.35 [0.29, 0.42] | 0.44 [0.38, 0.51] | 0.35 [0.29, 0.43] | -- | 0.08 (+23.4%) | -0.08 (-19.2%) | -- |

Clin Hx means clinical history. Δa represents the performance shift of the models between inputs with clinical history + Images vs. clinical history only. Δb represents the performance shift of the models between inputs with clinical history + Volumes at 2fps vs. clinical history + Images. Δc represents the performance shift between clinical history + Volumes at 10fps vs. clinical history + Images. A Differences (Δ) were calculated from unrounded values prior to rounding to two decimal places; accordingly, minor discrepancies may be observed when subtracting the rounded scores presented in this table.

**Table S2. Top-1 accuracy of models across input modalities and datasets**

| **Models** | **RadEdu** | | |  | **RealClin** | | | | | |  |
| --- | --- | --- | --- | --- | --- | --- | --- | --- | --- | --- | --- |
|  | **Clin Hx** | **Clin Hx + Images** | **Δa** |  | **Clin Hx** | **Clin Hx + Images** | **Clin Hx + Volumes (2fps)** | **Clin Hx + Volumes (10fps)** | **Δa** | **Δb** | **Δc** |
| **Proprietary MLLMs** | | | | | | | | | | |  |
| gemini-2-flash | 12.5 [9.2, 16.1] | 29.7 [25.0, 34.4] | 17.2 (+137.8%) |  | **18.9 [15.0, 23.1]** | **28.6 [23.9, 33.3]** | **23.9 [19.4, 28.3]** | **25.0 [20.6, 29.4]** | 9.7 (+51.5%) | -4.7 (-16.5%) | -3.6 (-12.6%) |
| gemini-2.5-flash-thinking | **16.1 [12.5, 20.0]** | 31.4 [26.7, 36.1] | 15.3 (+94.8%) |  | 17.2 [13.3, 21.1] | 26.1 [21.7, 30.8] | 18.6 [14.7, 22.8] | 17.5 [13.6, 21.4] | 8.9 (+51.6%) | -7.5 (-28.7%) | -8.6 (-33.0%) |
| gpt-4o | 13.6 [10.3, 17.2] | 23.6 [19.2, 28.1] | 10.0 (+73.5%) |  | 15.8 [12.2, 19.7] | 25.0 [20.6, 29.4] | 21.7 [17.5, 26.1] | 22.8 [18.6, 27.2] | 9.2 (+57.9%) | -3.3 (-13.3%) | -2.2 (-8.9%) |
| o3 | 14.2 [10.6, 17.8] | **34.4 [29.4, 39.4]** | 20.3 (+143.1%) |  | 17.8 [13.9, 21.9] | 22.2 [18.1, 26.7] | 18.1 [14.2, 22.2] | 21.7 [17.5, 26.1] | 4.4 (+25.0%) | -4.2 (-18.8%) | -0.6 (-2.5%) |
| **Large open-source MLLMs** | | | | | | | | | | |  |
| qwen2.5-vl-72b | 11.1 [8.1, 14.4] | **18.1 [14.2, 22.2]** | 6.9 (+62.5%) |  | **18.1 [14.2, 22.2]** | 25.0 [20.6, 29.4] | 21.1 [16.9, 25.3] | **20.6 [16.4, 24.7]** | 6.9 (+38.5%) | -3.9 (-15.6%) | -4.4 (-17.8%) |
| internvl3-78b | **11.4 [8.3, 14.7]** | 15.6 [11.9, 19.4] | 4.2 (+36.6%) |  | 16.7 [12.8, 20.6] | **26.1 [21.7, 30.8]** | **23.9 [19.4, 28.3]** | 19.7 [15.8, 23.9] | 9.4 (+56.7%) | -2.2 (-8.5%) | -6.4 (-24.5%) |
| llama-4-scout-17b-16e | 10.8 [7.8, 14.2] | 15.3 [11.7, 19.2] | 4.4 (+41.0%) |  | 11.1 [8.1, 14.4] | 20.3 [16.1, 24.4] | 12.5 [9.2, 16.1] | -- | 9.2 (+82.5%) | -7.8 (-38.4%) | -- |
| **Small open-source MLLMs** | | | | | | | | | | |  |
| qwen2.5-vl-7b | 5.6 [3.3, 8.1] | 7.8 [5.0, 10.6] | 2.2 (+40.0%) |  | 12.8 [9.4, 16.4] | 17.2 [13.3, 21.1] | 17.5 [13.6, 21.4] | 15.3 [11.7, 19.2] | 4.4 (+34.8%) | 0.3 (+1.6%) | -1.9 (-11.3%) |
| internvl3-8b | 5.0 [2.8, 7.2] | 7.8 [5.0, 10.6] | 2.8 (+55.6%) |  | **13.6 [10.3, 17.2]** | 18.6 [14.7, 22.8**]** | **18.1 [14.2, 22.2]** | **17.5 [13.6, 21.4]** | 5.0 (+36.7%) | -0.6 (-3.0%) | -1.1 (-6.0%) |
| medgemma-4b | **6.7 [4.2, 9.4]** | **8.3 [5.6, 11.4]** | 1.7 (+25.0%) |  | 12.8 [9.4, 16.4] | **22.5 [18.3, 26.9]** | 13.3 [10.0, 16.9] | -- | 9.7 (+76.1%) | -9.2 (-40.7%) | -- |

Clin Hx means clinical history. Δa represents the performance shift of the models between inputs with clinical history + Images vs. clinical history only. Δb represents the performance shift of the models between inputs with clinical history + Volumes at 2fps vs. clinical history + Images. Δc represents the performance shift between clinical history + Volumes at 10fps vs. clinical history + Images. A Differences (Δ) were calculated from unrounded values prior to rounding to two decimal places; accordingly, minor discrepancies may be observed when subtracting the rounded scores presented in this table.

**Table S3. Top-3 accuracy of models across input modalities and datasets**

| **Models** | **RadEdu** | | |  | **RealClin** | | | | | |  |
| --- | --- | --- | --- | --- | --- | --- | --- | --- | --- | --- | --- |
|  | **Clin Hx** | **Clin Hx + Images** | **Δa** |  | **Clin Hx** | **Clin Hx + Images** | **Clin Hx + Volumes (2fps)** | **Clin Hx + Volumes (10fps)** | **Δa** | **Δb** | **Δc** |
| **Proprietary MLLMs** | | | | | | | | | | |  |
| gemini-2-flash | 20.0 [15.8, 24.2] | 38.1 [33.1, 43.1] | 18.1 (+90.3%) |  | **29.2 [24.4, 33.9]** | **43.6 [38.6, 48.6]** | **38.1 [33.1, 43.1]** | 37.5 [32.5, 42.5] | 14.4 (+49.5%) | -5.6 (-12.7%) | -6.1 (-14.0%) |
| gemini-2.5-flash-thinking | **24.4 [20.0, 28.9]** | 42.8 [37.8, 47.8] | 18.3 (+75.0%) |  | 27.8 [23.3, 32.5] | 39.4 [34.4, 44.4] | 29.2 [24.4, 33.9] | 25.8 [21.4, 30.3] | 11.7 (+42.0%) | -10.3 (-26.1%) | -13.6 (-34.5%) |
| gpt-4o | 20.8 [16.7, 25.0] | 37.5 [32.5, 42.5] | 16.7 (+80.0%) |  | 28.6 [23.9, 33.3] | 40.6 [35.6, 45.6] | 37.2 [32.2, 42.2] | **37.8 [32.8, 42.8]** | 11.9 (+41.7%) | -3.3 (-8.2%) | -2.8 (-6.8%) |
| o3 | 20.3 [16.1, 24.4] | **46.7 [41.4, 51.9]** | 26.4 (+130.1%) |  | 28.3 [23.6, 33.1] | 33.9 [29.2, 38.9] | 30.3 [25.6, 35.0] | 32.8 [28.1, 37.8] | 5.6 (+19.6%) | -3.6 (-10.7%) | -1.1 (-3.3%) |
| **Large open-source MLLMs** | | | | | | | | | | |  |
| qwen2.5-vl-72b | 16.1 [12.5, 20.0] | **27.5 [23.1, 32.2]** | 11.4 (+70.7%) |  | **29.7 [25.0, 34.4]** | 36.9 [31.9, 41.9] | 34.2 [29.4, 39.2] | **34.2 [29.4, 39.2]** | 7.2 (+24.3%) | -2.8 (-7.5%) | -2.8 (-7.5%) |
| internvl3-78b | 16.7 [12.8, 20.6] | 23.1 [18.9, 27.5] | 6.4 (+38.3%) |  | 27.2 [22.8, 31.9] | **39.4 [34.4, 44.4]** | **37.8 [32.8, 42.8]** | 32.8 [28.1, 37.8] | 12.2 (+44.9%) | -1.7 (-4.2%) | -6.7 (-16.9%) |
| llama-4-scout-17b-16e | **17.2 [13.3, 21.1]** | 22.5 [18.3, 26.9] | 5.3 (+30.6%) |  | 24.2 [19.7, 28.6] | 30.6 [25.8, 35.3] | 23.6 [19.2, 28.1] | -- | 6.4 (+26.4%) | -6.9 (-22.7%) | -- |
| **Small open-source MLLMs** | | | | | | | | | | |  |
| qwen2.5-vl-7b | 9.2 [6.4, 12.2] | 12.5 [9.2, 16.1] | 3.3 (+36.4%) |  | **23.3 [19.2, 27.8]** | 30.6 [25.8, 35.3] | 31.4 [26.7, 36.1] | **29.7 [25.0, 34.4]** | 7.2 (+31.0%) | 0.8 (+2.7%) | -0.8 (-2.7%) |
| internvl3-8b | 10.3 [7.2, 13.6] | 12.5 [9.2, 16.1] | 2.2 (+21.6%) |  | 23.1 [18.9, 27.5] | 33.3 [28.6, 38.3] | **35.3 [30.3, 40.3]** | **29.7 [25.0, 34.4]** | 10.3 (+44.6%) | 1.9 (+5.8%) | -3.6 (-10.8%) |
| medgemma-4b | **13.3 [10.0, 16.9]** | **15.0 [11.4, 18.9]** | 1.7 (+12.5%) |  | 22.8 [18.6, 27.2] | **33.9 [29.2, 38.9]** | 22.5 [18.3, 26.9] | -- | 11.1 (+48.8%) | -11.4 (-33.6%) | -- |

Clin Hx means clinical history. Δa represents the performance shift of the models between inputs with clinical history + Images vs. clinical history only. Δb represents the performance shift of the models between inputs with clinical history + Volumes at 2fps vs. clinical history + Images. Δc represents the performance shift between clinical history + Volumes at 10fps vs. clinical history + Images. A Differences (Δ) were calculated from unrounded values prior to rounding to two decimal places; accordingly, minor discrepancies may be observed when subtracting the rounded scores presented in this table.

**Table S4. Top-5 accuracy of models across input modalities and datasets**

| **Models** | **RadEdu** | | |  | **RealClin** | | | | | |  |
| --- | --- | --- | --- | --- | --- | --- | --- | --- | --- | --- | --- |
|  | **Clin Hx** | **Clin Hx + Images** | **Δa** |  | **Clin Hx** | **Clin Hx + Images** | **Clin Hx + Volumes (2fps)** | **Clin Hx + Volumes (10fps)** | **Δa** | **Δb** | **Δc** |
| **Proprietary MLLMs** | | | | | | | | | | |  |
| gemini-2-flash | 24.7 [20.3, 29.2] | 42.5 [37.5, 47.5] | 17.8 (+71.9%) |  | **37.8 [32.8, 42.8]** | **48.9 [43.6, 54.2]** | 42.5 [37.5, 47.5] | 42.2 [37.2, 47.2] | 11.1 (+29.4%) | -6.4 (-13.1%) | -6.7 (-13.6%) |
| gemini-2.5-flash-thinking | **27.8 [23.3, 32.5]** | 46.9 [41.7, 52.2] | 19.2 (+69.0%) |  | 32.5 [27.8, 37.5] | 44.4 [39.4, 49.7] | 33.3 [28.6, 38.3] | 31.9 [27.2, 36.7] | 11.9 (+36.8%) | -11.1 (-25.0%) | -12.5 (-28.1%) |
| gpt-4o | 23.9 [19.4, 28.3] | 42.2 [37.2, 47.2] | 18.3 (+76.7%) |  | **37.8 [32.8, 42.8]** | 48.1 [42.8, 53.3] | **46.9 [41.7, 52.2]** | **45.0 [40.0, 50.3]** | 10.3 (+27.2%) | -1.1 (-2.3%) | -3.1 (-6.4%) |
| o3 | 23.3 [19.2, 27.8] | **48.6 [43.3, 53.9]** | 25.3 (+108.3%) |  | 34.2 [29.4, 39.2] | 41.1 [36.1, 46.1] | 36.9 [31.9, 41.9] | 37.5 [32.5, 42.5] | 6.9 (+20.3%) | -4.2 (-10.1%) | -3.6 (-8.8%) |
| **Large open-source MLLMs** | | | | | | | | | | |  |
| qwen2.5-vl-72b | 19.7 [15.8, 23.9] | **30.6 [25.8, 35.3]** | 10.8 (+54.9%) |  | **35.6 [30.6, 40.6]** | 44.2 [39.2, 49.4] | 40.3 [35.3, 45.3] | 38.9 [33.9, 43.9] | 8.6 (+24.2%) | -3.9 (-8.8%) | -5.3 (-11.9%) |
| internvl3-78b | **20.3 [16.1, 24.4]** | 26.4 [21.9, 31.1] | 6.11 (+30.1%) |  | 34.7 [29.7, 39.7] | **48.1 [42.8, 53.3]** | **45.8 [40.8, 51.1]** | **41.1 [36.1, 46.1]** | 13.3 (+38.4%) | -2.2 (-4.6%) | -6.9 (-14.5%) |
| llama-4-scout-17b-16e | **20.3 [16.1, 24.4]** | 25.6 [21.1, 30.0] | 5.3 (+26.0%) |  | 31.7 [26.9, 36.4] | 37.2 [32.2, 42.2] | 32.8 [28.1, 37.8] | -- | 5.6 (+17.5%) | -4.4 (-11.9%) | -- |
| **Small open-source MLLMs** | | | | | | | | | | |  |
| qwen2.5-vl-7b | 11.1 [8.1, 14.4] | 15.3 [11.7, 19.2] | 4.2 (+37.5%) |  | 30.6 [25.8, 35.3] | 37.8 [32.8, 42.8] | 39.4 [34.4, 44.4] | 36.7 [31.7, 41.7] | 7.2 (+23.6%) | 1.7 (+4.4%) | -1.1 (-2.9%) |
| internvl3-8b | 11.9 [8.6, 15.3] | 15.3 [11.7, 19.2] | 3.3 (+27.9%) |  | **33.6 [28.9, 38.6]** | **39.2 [34.2, 44.2]** | **41.9 [36.9, 46.9]** | **37.8 [32.8, 42.8]** | 5.6 (+16.5%) | 2.8 (+7.1%) | -1.4 (-3.5%) |
| medgemma-4b | **15.8 [12.2, 19.7]** | **17.2 [13.3, 21.1]** | 1.9 (+12.7%) |  | 33.3 [28.6, 38.3] | **39.2 [34.2, 44.2]** | 33.6 [28.9, 38.6] | -- | 5.8 (+17.5%) | -5.6 (-14.2%) | -- |

Clin Hx means clinical history. Δa represents the performance shift of the models between inputs with clinical history + Images vs. clinical history only. Δb represents the performance shift of the models between inputs with clinical history + Volumes at 2fps vs. clinical history + Images. Δc represents the performance shift between clinical history + Volumes at 10fps vs. clinical history + Images. A Differences (Δ) were calculated from unrounded values prior to rounding to two decimal places; accordingly, minor discrepancies may be observed when subtracting the rounded scores presented in this table.

**Table S5. Performance transition across input modalities in RealClin dataset**

| **Models** | **Clin Hx —> Clin Hx + Images** | | |  | **Clin Hx + Images —> Clin Hx + Volumes (2 fps)** | | |  | **Clin Hx + Images —> Clin Hx + Volumes (10 fps)** | | |
| --- | --- | --- | --- | --- | --- | --- | --- | --- | --- | --- | --- |
|  | Improve | Maintain | Decline |  | Improve | Maintain | Decline |  | Improve | Maintain | Decline |
| gpt4o | 74 (20.6%) | 266 (73.9%) | 20 (5.6%) |  | 24 (6.7%) | 296 (82.2%) | 40 (11.1%) |  | 110 (30.6%) | 121 (33.6%) | 129 (35.8%) |
| o3 | 73 (20.3%) | 247 (68.6%) | 40 (11.1%) |  | 47 (13.1%) | 254 (70.6%) | 59 (16.4%) |  | 42 (11.7%) | 261 (72.5%) | 57 (15.8%) |
| gemini-2-flash | 100 (27.8%) | 220 (61.1%) | 40 (11.1%) |  | 86 (23.9%) | 161 (44.7%) | 113 (31.4%) |  | 58 (16.1%) | 222 (61.7%) | 80 (22.2%) |
| gemini-2.5-flash-thinking | 94 (26.1%) | 222 (61.7%) | 44 (12.2%) |  | 39 (10.8%) | 248 (68.9%) | 73 (20.3%) |  | 35 (9.7%) | 245 (68.1%) | 80 (22.2%) |
| qwen2.5-vl-72b | 72 (20.0%) | 261 (72.5%) | 27 (7.5%) |  | 22 (6.1%) | 296 (82.2%) | 42 (11.7%) |  | 20 (5.6%) | 297 (82.5%) | 43 (11.9%) |
| internvl3-78b | 73 (20.3%) | 271 (75.3%) | 16 (4.4%) |  | 21 (5.8%) | 310 (86.1%) | 29 (8.1%) |  | 21 (5.8%) | 278 (77.2%) | 61 (16.9%) |

Clin Hx means clinical history.

**Table S6. Performance shift of models in RealClin after translating clinical history from Chinese into English**

| **Models** | **Clin Hx** | **Clin Hx translation** | **Δa** | **W** | ***P*** | ***Padj*** |
| --- | --- | --- | --- | --- | --- | --- |
| **Proprietary MLLMs** | | | |  |  |  |
| gemini-2-flash | 0.52 [0.45, 0.61] | 0.44 [0.36, 0.53] | -0.08 | 3009.5 | .03 | .03 |
| gemini-2.5-flash-thinking | 0.54 [0.45, 0.63] | 0.44 [0.35, 0.52] | -0.10 | 1678.0 | .01 | .02 |
| gpt-4o | 0.54 [0.46, 0.63] | 0.44 [0.36, 0.52] | -0.10 | 2993.5 | .02 | .03 |
| o3 | 0.59 [0.49, 0.68] | 0.40 [0.32, 0.48] | -0.19 | 1232.0 | <.001 | <.001 |
| **Large open-source MLLMs** | | | |  |  |  |
| qwen2.5-vl-72b | 0.51 [0.43, 0.59] | 0.33 [0.26, 0.40] | -0.18 | 2121.5 | <.001 | <.001 |
| internvl3-78b | 0.52 [0.44, 0.62] | 0.36 [0.29, 0.44] | -0.16 | 2352.5 | <.001 | <.001 |
| llama-4-scout-17b-16e | 0.42 [0.35, 0.50] | 0.39 [0.32, 0.47] | -0.03 | 3239.5 | .38 | .38 |
| **Small open-source MLLMs** | | | |  |  |  |
| qwen2.5-vl-7b | 0.39 [0.32, 0.47] | 0.26 [0.20, 0.33] | -0.13 | 2478.5 | <.001 | <.001 |
| internvl3-8b | 0.42 [0.35, 0.49] | 0.29 [0.23, 0.36] | -0.13 | 2447.5 | .001 | .002 |
| medgemma-4b | 0.35 [0.29, 0.42] | 0.16 [0.11, 0.22] | -0.19 | 1828.5 | <.001 | <.001 |

Clin Hx means clinical history. Δa represents performance shift of models in RealClin after translating clinical history from Chinese into English. *Padj* means the adjusted P-values using the Benjamini-Hochberg procedure with q=.05. A Differences (Δ) were calculated from unrounded values prior to rounding to two decimal places; accordingly, minor discrepancies may be observed when subtracting the rounded scores presented in this table.

**Table S7. Impact of disease rarity on cross-dataset performance**

| **Models** | **Disease Rarity** | **RadEdu** | | |  | **RealClin** | | |
| --- | --- | --- | --- | --- | --- | --- | --- | --- |
|  |  | **Clin Hx** | **Clin Hx + Images** | **Δa** |  | **Clin Hx** | **Clin Hx + Images** | **Δa** |
| **Proprietary MLLMs** |  |  |  |  |  |  |  |  |
| gemini-2-flash | Common | 0.38 [0.28, 0.51] | 0.77 [0.62, 0.93] | 0.39 (+101.3%) |  | 0.53 [0.45, 0.63] | 0.80 [0.69, 0.90] | 0.26 (+49.0%) |
| gemini-2-flash | Rare | 0.48 [0.36, 0.63] | 0.88 [0.73, 1.06] | 0.40 (+81.9%) |  | 0.41 [0.21, 0.70] | 1.10 [0.72, 1.48] | 0.69 (+167.2%) |
| gemini-2.5-flash-thinking | Common | 0.43 [0.32, 0.56] | 0.84 [0.69, 0.99] | 0.41 (+95.9%) |  | 0.56 [0.47, 0.65] | 0.76 [0.65, 0.87] | 0.20 (+36.0%) |
| gemini-2.5-flash-thinking | Rare | 0.62 [0.47, 0.77] | 1.04 [0.87, 1.22] | 0.42 (+67.1%) |  | 0.32 [0.13, 0.66] | 1.20 [0.83, 1.57] | 0.88 (+272.0%) |
| gpt4o | Common | 0.37 [0.26, 0.51] | 0.77 [0.63, 0.94] | 0.40 (+107.4%) |  | 0.54 [0.45, 0.63] | 0.75 [0.65, 0.85] | 0.21 (+39.1%) |
| gpt4o | Rare | 0.51 [0.38, 0.65] | 0.84 [0.69, 1.00] | 0.34 (+66.7%) |  | 0.57 [0.30, 0.88] | 1.01 [0.68, 1.40] | 0.45 (+78.4%) |
| o3 | Common | 0.37 [0.27, 0.51] | 1.04 [0.87, 1.22] | 0.67 (+182.3%) |  | 0.58 [0.49, 0.69] | 0.76 [0.65, 0.87] | 0.17 (+29.7%) |
| o3 | Rare | 0.60 [0.46, 0.76] | 1.26 [1.06, 1.45] | 0.65 (+107.7%) |  | 0.61 [0.34, 0.94] | 1.15 [0.78, 1.54] | 0.55 (+90.4%) |
| **Large open-source MLLMs** | |  |  |  |  |  |  |  |
| qwen2.5-vl-72b | Common | 0.31 [0.21, 0.43] | 0.53 [0.40, 0.67] | 0.22 (+69.7%) |  | 0.52 [0.44, 0.61] | 0.71 [0.62, 0.82] | 0.19 (+35.3%) |
| qwen2.5-vl-72b | Rare | 0.38 [0.27, 0.52] | 0.67 [0.52, 0.82] | 0.29 (+76.1%) |  | 0.33 [0.15, 0.59] | 0.67 [0.38, 1.03] | 0.34 (+103.9%) |
| internvl3-78b | Common | 0.29 [0.19, 0.40] | 0.43 [0.31, 0.56] | 0.14 (+50.4%) |  | 0.53 [0.44, 0.63] | 0.76 [0.66, 0.86] | 0.23 (+43.1%) |
| internvl3-78b | Rare | 0.41 [0.30, 0.54] | 0.53 [0.41, 0.68] | 0.13 (+31.2%) |  | 0.44 [0.23, 0.76] | 0.67 [0.37, 0.99] | 0.23 (+52.9%) |
| llama-4-scout-17b-16e | Common | 0.29 [0.19, 0.42] | 0.40 [0.29, 0.53] | 0.11 (+36.0%) |  | 0.42 [0.34, 0.50] | 0.56 [0.47, 0.66] | 0.15 (+35.5%) |
| llama-4-scout-17b-16e | Rare | 0.45 [0.33, 0.59] | 0.58 [0.44, 0.73] | 0.13 (+28.9%) |  | 0.43 [0.22, 0.77] | 0.92 [0.59, 1.31] | 0.49 (+115.2%) |
| **Small open-source MLLMs** | |  |  |  |  |  |  |  |
| qwen2.5-vl-7b | Common | 0.15 [0.09, 0.24] | 0.21 [0.13, 0.31] | 0.06 (+36.9%) |  | 0.41 [0.34, 0.50] | 0.53 [0.45, 0.62] | 0.11 (+27.4%) |
| qwen2.5-vl-7b | Rare | 0.20 [0.13, 0.31] | 0.28 [0.19, 0.40] | 0.07 (+36.3%) |  | 0.11 [0.03, 0.32] | 0.19 [0.06, 0.46] | 0.08 (+70.6%) |
| internvl3-8b | Common | 0.16 [0.09, 0.25] | 0.19 [0.12, 0.30] | 0.04 (+23.0%) |  | 0.42 [0.34, 0.51] | 0.56 [0.48, 0.65] | 0.14 (+32.3%) |
| internvl3-8b | Rare | 0.25 [0.16, 0.38] | 0.33 [0.22, 0.47] | 0.08 (+30.1%) |  | 0.36 [0.17, 0.66] | 0.55 [0.31, 0.85] | 0.19 (+51.8%) |
| medgemma-4b | Common | 0.26 [0.17, 0.37] | 0.25 [0.17, 0.36] | -0.01 (-3.6%) |  | 0.38 [0.32, 0.45] | 0.46 [0.39, 0.53] | 0.08 (+21.4%) |
| medgemma-4b | Rare | 0.23 [0.15, 0.34] | 0.35 [0.25, 0.47] | 0.12 (+50.8%) |  | 0.10 [0.03, 0.23] | 0.20 [0.08, 0.35] | 0.10 (+106.7%) |

Clin Hx means clinical history. Δa represents the performance shift of the models between inputs with clinical history + Images vs. clinical history only. A Differences (Δ) were calculated from unrounded values prior to rounding to two decimal places; accordingly, minor discrepancies may be observed when subtracting the rounded scores presented in this table.
